# The effectiveness of peer-led interventions to improve work-related psychosocial outcomes and reduce turnover of support workers in residential aged care: A systematic review and meta-analysis

**DOI:** 10.1016/j.ijnsa.2023.100158

**Published:** 2023-10-15

**Authors:** Karol J. Czuba, Alain C. Vandal, Frances M. Czuba, Nicola M. Kayes

**Affiliations:** a Centre for Person Centred Research, Auckland University of Technology, Northcote, Auckland 0627, New Zealand; b Department of Biostatistics and Epidemiology, Faculty of Health and Environmental Sciences, Auckland University of Technology, Auckland, New Zealand; c Whangarei Hospital, Private Bag 9742, Whangārei 0148, New Zealand

**Keywords:** Carers, Residential facilities, Homes for the aged, Systematic review, Occupational stress, Meta-analysis, Personnel turnover, Job satisfaction

## Abstract

**Background:**

Support workers are central to the delivery of residential aged care, but the workforce is facing increasing work demands and widespread shortages. This contributes to high rates of burnout, decreased job satisfaction and high staff turnover. Peer-led interventions are reported to be effective but it is necessary to use evidence-based interventions to support this key workforce group.

**Objective:**

This study aimed to evaluate the scientific evidence on effectiveness of strategies improving psychosocial and turnover-related outcomes for support workers in aged care that could be incorporated into a peer-led intervention.

**Design:**

Systematic review and meta-analyses of experimental and quasi-experimental studies.

**Setting:**

Residential aged care.

**Methods:**

A systematic literature review was conducted using MEDLINE (via PubMed), EMBASE (via Scopus), and CINAHL (via EBSCO). We included studies examining the effectiveness of workplace interventions aiming to reduce aged care support workers' turnover rates and/or improve their work-related psychosocial outcomes (such as work stress, job satisfaction, self-esteem, and other). A number of meta-analyses using a mixed-effects model were performed to calculate standardized mean differences and odds ratios.

**Results:**

Fifty-one studies were included: 15 randomised controlled trials (RCTs), 19 non-RCTs and 17 Pre-Post studies. Most of the studies were rated as having ‘high’ or ‘very high risk of bias’. The studies were clustered by intervention type: 1) knowledge-based, 2) interpersonal skills-based, 3) team-building, and 4) self-care. Knowledge-based interventions were the most used approach, with 26 studies in this category, and frequently reported improvements in stress- and satisfaction-related outcomes. There were twelve interpersonal skills-based and nine team-building interventions, which often reported decreased work stress, staff turnover, and intention to quit. There were four self-care interventions of which only one reported improvements in stress-related outcomes. Meta-analyses showed that only knowledge-based interventions resulted in statistically significant improvements: lower staff turnover rates (OR 0.47, 95 %CI: 0.37, 060), and higher scores for job/life satisfaction (SMD 0.26, 95 % CI: 0.05, 0.46) and staff attitude (SMD 0.23, 95 % CI: 0.05, 0.45).

**Conclusion:**

This review found numerous strategies that have been trialled to improve support workers’ psychosocial- and turnover-related outcomes. Most studies reported improvements in outcomes. However, our meta-analyses suggest that the effect sizes were small and mostly non-significant, with the evidence being of low certainty. The evidence for effectiveness of knowledge-based interventions appears the most convincing, with statistically significant improvements reported for turnover rates, job/life satisfaction and staff attitude. More high-quality studies are needed to consolidate the existing evidence.

**PROSPERO registration number:**

CRD42017059007; 02 June 2017.

Tweetable abstract: Knowledge-based interventions most promising in improving support workers’ outcomes in aged care. #agedcare #staffturnover

## What is already known about the topic?

• Support workers in New Zealand, and worldwide, are a key workforce in the provision of residential aged care.
• Their work performance and well-being are challenged by increasingly high workloads and poor working conditions, affecting their wellbeing and the quality of care they provide.
• Support workers need to be supported better when facing increased workloads and poor working conditions.

## What this paper adds

• Knowledge-based interventions were found to be the most studied approach to improve support workers’ turnover-related and psychosocial outcomes.
• Knowledge-based interventions were found to lead to small statistically significant reductions in staff turnover, and improvements in job/life satisfaction and staff attitude.
• Overall, the certainty of evidence is low and marked by study designs’ heterogeneity. More high-quality studies are needed to consolidate the evidence.

## 1 Background

Support workers play a vital role in the delivery of residential aged care (Australian Government Department of Health, 2021; Bird et al., 2015). They are known by many titles, for example formal caregivers, healthcare aides, nursing assistants or personal care attendants. They make up a large proportion of the long-term care workforce, over 60 % in UK (Health Education England, 2015) and over 70 % in Australia (Australian Government Department of Health, 2021). Broadly defined, their main duty is to assist older people with activities of daily living, which includes assisting with personal cares, feeding, cleaning and laundry, rehabilitation, and independence (Ravenswood et al., 2014). However, the role of support workers extends beyond practical tasks. It also includes spiritual aspects of caring pertaining to establishing and maintaining psychosocial relationships with the residents (Touhy, 2004; Yoder, 2010). Their performance plays a crucial role in the quality of care that people in long-term care receive (Bird et al., 2015).

Globally, the demand for aged care already exceeds the supply of available workers (Ravenswood et al., 2014; Royal Commission into Aged Care Quality and Safety, 2020; Huang et al., 2023) contributing to increased workload for this group, as well as heightened occupational stress (Dawson et al., 2014; Halpin et al., 2017). Consequently, this can lead to adverse changes in a range of priority outcomes: increased staff turnover rates (Gao et al., 2017; Jurij et al., 2023), decreased job satisfaction (Hodgkin et al., 2017; Xiao et al., 2021), and decreased perceived health and well-being (Perry et al., 2017; Orrung Wallin et al., 2015). The demand for aged care and workforce shortages are predicted to further increase (Austin et al., 2021; Huang et al., 2023; World Health Organization, 2015). It is necessary to tackle the abovementioned challenges and regressions in the priority outcomes with effective, evidence-based interventions. Given the abovementioned constraints, such interventions may have to be delivered within the existing structures, and with minimal training and ongoing costs.

One approach, which has been shown to improve the priority outcomes for this workforce, is training a dedicated support worker to coordinate the delivery of a range of support strategies to their co-workers (Hewko et al., 2015). Interventions using such an approach are often built around the concept of peer-mentoring (Dill et al., 2010; Pillemer et al., 2008; Rausch, 2016; Resnick et al., 2009), where more experienced support workers are trained to work with less experienced peers, identify their needs and coordinate specific support actions to address these needs. However, the rationale for selecting the specific interventions delivered by peer-mentors has not been explicit in existing research. Further, while the interventions were effective in improving some outcomes (for example, staff turnover), other outcomes were not well addressed (for example, job satisfaction). A systematic literature review could help identifying strategies to include in a peer-led intervention to improve the key outcomes for this workforce.

The primary aim of this systematic review was to evaluate the scientific evidence on the effectiveness of interventions that could be peer-led in improving psychosocial and turnover-related outcomes for support workers in aged care. To the best of our knowledge, no existing systematic review or meta-analysis addressing this aim has been published. Findings from this review would aid aged care providers in selecting interventions that are effective and applicable to their workforce, as well as guide further research into areas with inconclusive findings.

## 2 Methods

### 2.1 Protocol and registration

The review protocol was registered and published with PROSPERO (Czuba et al., 2017); registration number: CRD42017059007; 02 June 2017).

### 2.2 Eligibility criteria

Studies evaluating effectiveness of interventions were included. This review was carried out to inform the direction of the larger project, and it included a range of experimental and quasi-experimental designs: randomised controlled trials [RCT], nonrandomised controlled trials [nRCT], and single-arm pre-post studies [Pre-Post]. Multiple publications reporting on the same study were counted as a single study. Only studies published in English were eligible. No restrictions were placed on date or study location.

Studies were included if: a) they were conducted in a long-term aged care facility setting (for example nursing home or rest home); b) they included support workers as the primary population of interest; c) the intervention or strategy being tested could reasonably be peer-led; and d) they were evaluating workplace interventions (individual-based and organisation-based) aiming to improve support workers’ work-related psychosocial and/or turnover-related outcomes.

Studies were excluded if the perceived cost of implementation would likely preclude uptake in the current financially constrained context (e.g., interventions requiring a system-level change, such as facility restructuring).

We defined psychosocial outcomes as a person's psychological state related to their social interactions at the workplace (Czuba et al., 2017). ‘Turnover-related’ outcomes were defined as any outcome related to an employee failing to show up at work, such as turnover, retention, intention to quit, absenteeism, and similar (Czuba et al., 2017).

### 2.3 Search strategy

We systematically searched the following databases for articles published before 6 March 2023: MEDLINE (via PubMed), EMBASE (via Scopus), and CINAHL (via EBSCO). In addition, the British Journal of Healthcare Assistants was searched as it is the only journal dedicated solely to publishing research related to this workforce. The search strategy included terms related to:

1. Population: Aged care support workers – search terms included: support workers, nursing assistants, caregivers, and other.
2. Outcome: Turnover and work-related psychosocial outcomes - search terms included: turnover, retention, job satisfaction, quality of work life, stress, and other.
3. Setting: Long-term aged care facility - search terms included nursing home, rest home, and other.

The detailed search strategies are presented in Supplementary file: Appendix 1.

### 2.4 Study selection

Titles and abstracts returned using the above search strategy were screened for relevance. The titles and abstracts retrieved were screened by Karol Czuba (KC). A random sample of 50 titles and abstracts were screened independently by Frances Czuba. Interrater agreement between both reviewers’ inclusion and exclusion decisions was 100 %. Full texts of potentially eligible studies were then retrieved and independently assessed by KC. Where the eligibility was unclear, this was discussed and resolved with co-authors.

### 2.5 Data extraction

To develop an initial description of findings from the included studies, a data extraction table was created. Data about the design, country, setting, participants, intervention, and study outcomes were extracted by KC. Missing data were requested from study authors, with eight authors providing additional data.

### 2.6 Risk of bias assessment

All papers selected for the main review were assessed for risk of bias using the relevant Critical Appraisal Checklist from the Scottish Intercollegiate Guidelines Network (Scottish Intercollegiate Guidelines Network, 2007). The categories proposed by the Checklist's authors were relabelled to reflect the specific focus on the risk of bias level:

- ‘High quality’ to ‘low risk’
- ‘Acceptable’ to ‘moderate risk’
- ‘Low quality’ to ‘high risk’
- ‘Unacceptable’ to ‘very high risk’

Each study was assessed by KC and placed into a risk of bias category – low, moderate, high, or very high. All studies using single-arm pre-post designs were rated ‘very high risk of bias’ in line with the Cochrane Collaboration's guidelines for systematic reviews of interventions (Higgins and Green, 2011).

### 2.7 Data synthesis and analysis

All interventions were evaluated for their key components, including specific intervention activities (e.g., teaching communication skills), and structural (e.g., intervention duration) and contextual (e.g., intervention setting) factors. To manage the high heterogeneity, we clustered the studies into four groups by intervention type. All studies were then grouped and clustered by intervention type and study design. Each study's intervention type was determined by its main component/s (Supplementary file: Appendix 2) and the primary focus of the intervention. Summary tables were created for each intervention type (Supplementary file: Tables 1–4). The four intervention types were:

1. Knowledge-based interventions (26 studies) – interventions focusing primarily on enhancing staff knowledge and understanding of issues related to provision of aged care and ageing (e.g., education sessions and materials).
2. Interpersonal skills-based interventions (12 studies) – interventions focusing primarily on enhancing staff's interpersonal skills (e.g., interpersonal skills training), especially in interactions with other staff members or residents.
3. Team-building interventions (9 studies) – interventions focusing primarily on empowering the staff through facilitating their teamwork (e.g., involving them in team meetings or in decision making).
4. Self-care interventions (4 studies) – interventions focusing primarily on encouraging and supporting active engagement in self-care practice, such as exercise, healthy diet, meditation.

Furthermore, over 35 staff outcomes were identified in the included studies, which were combined into 13 overarching outcome types (Supplementary file: Appendix 3). The results for job satisfaction and life satisfaction were combined, as they are strongly related conceptually and can predict one another (Unanue et al., 2017).

To combine data on effectiveness of the proposed interventions, a meta-analysis was conducted. We used RevMan 5.3 (The Cochrane Collaboration, 2014) to calculate standardised mean difference (SMD) for continuous outcomes, and odds ratio (OR) for dichotomous outcomes, using mixed-effects models. Where specific outcomes were measured across multiple time points, the last reported result for that outcome was used. Meta-analyses were conducted when there were at least two studies reporting the specific outcome. Heterogeneity was assessed through consideration of studies’ population and comparator groups, interventions’ components and duration, and also with the Chi-squared test and *I*^2^ statistic, with consideration for effect magnitude and direction. A Chi-squared test with *p*-value < 0.10 suggests presence of heterogeneity (Higgins and Green, 2011). The *I*^2^ statistic (the percentage of variation across studies that is due to between-study heterogeneity as opposed to chance) was used to assess the extent of heterogeneity (0 %–25 %, low; 26 %–50 %, moderate; over 50 %, high) (Halpin et al., 2017). No baseline-adjustments were performed. When an outcome measure was used with scores incrementing in the opposite direction than on the other commonly used measures for that type of outcome, the scores were multiplied by –1. The threshold for statistical significance was set at 5 % against two-sided alternatives, with no correction for multiple testing. Effect sizes were interpreted using Chen et al. guidelines (Chen et al., 2010). For OR, baseline probabilities were pooled across studies.

#### 2.7.1 Subgroup analyses

We assessed the impact of different follow-up duration: short-term (<3 months post-implementation), medium-term (3–12 months post-implementation), and long-term (>12 months post-implementation).

#### 2.7.2 Sensitivity analyses

Sensitivity analyses were performed with respect to study design (RCT, nRCT or Pre-Post) and comparator type (placebo vs non-placebo). Provision of no intervention or a waitlist control group were defined as non-placebo control groups.

## 3 Results

**Fig. 1:**
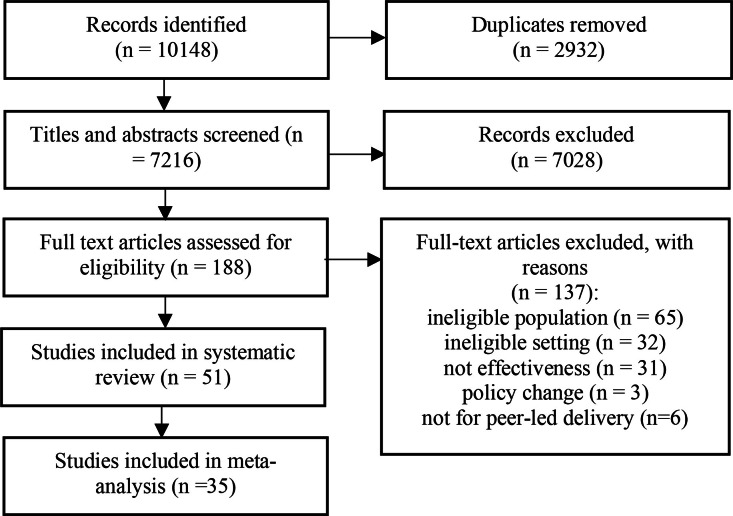
PRISMA flow chart.

Our initial search yielded 10,148 records. After duplicates were removed and screening was completed, 51 studies from 50 publications were included (Fig. 1; Supplementary file: Table 5); including 15 RCTs, 19 nRCTs and 17 Pre-Post studies.

Some of the most common exclusion reasons were: studies about family caregivers, studies about care recipients, studies not conducted in an aged care setting, qualitative studies, survey studies, editorials. The results are discussed by intervention type in the below.

### 3.1 Knowledge-based interventions

Twenty-six studies evaluated knowledge-based interventions (mean study sample size was *n* = 191 interquartile range (IQR) = 253; *n* = 4 studies did not report the sample size; Supplementary file: Table 5). Only one study was rated ‘low risk of bias’, and five studies were rated ‘moderate risk of bias’. The remaining studies were rated ‘high’ or ‘very high’ risk of bias (*n* = 9 and *n* = 11, respectively; Supplementary file: Table 5). The most common issues affecting the risk of bias in the included studies were lack of randomisation, inadequate concealment methods, lack of blinding and baseline differences between study groups.

Ten studies focused exclusively on support workers. Other studies either included other health staff groups (for example registered nurses; *n* = 13) or did not provide enough information to determine what the participants’ actual work roles were (for example, “study included professional caregivers”; *n* = 3). Eleven studies used a Pre-Post design, nine studies used a nRCT design, and six were RCTs. Twelve studies were conducted in the USA, three in Taiwan, two in Canada, two in Germany, and one each in the following: Brazil, Italy, Japan, Netherlands, Portugal, South Korea and Sweden. The most common settings were nursing homes (*n* = 11) and long-term care facilities (*n* = 11), followed by residential aged care facilities (*n* = 2), a special dementia care unit (*n* = 1) and a mixed setting of long-term care facility with in-home care (*n* = 1). Intervention duration varied between two weeks and 18 months, with a median of two months. Six studies did not report the duration of intervention.

Thirteen studies tested interventions using development of staff's knowledge (education) as the core intervention component. The remaining studies tested interventions using a combination of components: ‘education’ with ‘mentoring’ (*n* = 6), ‘education’ with ‘interpersonal skills’ (*n* = 4), ‘education’ with ‘mentoring’ and ‘relaxation, exercise and diet’ (*n* = 1), ‘education’ with ‘mentoring’ and ‘interpersonal skills’ (*n* = 1), and ‘education’ with ‘mentoring’ and ‘rewards’ (*n* = 1). The most common focus was on improving staff knowledge of dementia and ageing (*n* = 11). Four studies focused on general long-term care knowledge. Other foci included understanding and responding to work-related stress issues, education about empowerment, safe work environment, empathy, and a range of care approaches/philosophies (e.g., Person-Centred Care). Three studies reported testing interventions underpinned by Person-Centred Care approaches, and another three by self-efficacy theory; with a range of theories being reported by the remaining 13 studies). Seven studies did not report their interventions to be underpinned by a theoretical model. All studies tested new interventions.

#### 3.1.1 Knowledge-based interventions meta-analyses

The included studies evaluated 13 types of psychosocial and turnover-related outcomes. The most reported outcomes were stress-related outcomes (*n* = 15) and job/life satisfaction (*n* = 12). For the full list of reported outcomes please see Supplementary file: Appendix 3. Due to no data being available for meta-analysis or fewer than two studies in the comparison group, only seven types of outcomes were analysed (Table 1) and are reported below.

**Table 1:** Summary of effect sizes for each outcome comparison for knowledge-based interventions (statistically significant effect sizes bolded and are marked with ‘*’).

Outcome	Number of studies	Number of participants	Odds ratio or SMD [95 % CI]	*I*^2^ (heterogeneity)
**Turnover (OR)**				
All studies	2	769	*0.47 [0.37, 0.60]	0 %, *p* = 0.52
RCT	0	–	–	–
NRCT	1	16	021 [0.02, 2.52]	NA
Pre-Post	1	753	*0.48 [0.38, 0.60]	NA
Placebo	0	–	–	–
Non-placebo	1	16	0.21 [0.02, 2.52]	NA
Short-term follow-up	1	16	0.21 [0.02, 2.52]	NA
Medium-term follow-up	1	753	*0.48 [0.38, 0.60]	NA
Long-term follow-up	0	–	–	–
**Absenteeism (SMD)**				
All studies	2	151	−0.36 [−1.01, 0.29]	73 %, *p* = 0.05
RCT	2	151	−0.36 [−1.01, 0.29]	73 %, *p* = 0.05
NRCT	0	–	–	–
Pre-Post	0	–	–	–
Placebo	1	93	−0.06 [−0.46, 0.35]	NA
Non-placebo	1	58	*−0.72 [−1.26, −0.19]	NA
Short-term follow-up	1	58	*−0.72 [−1.26, −0.19]	NA
Medium-term follow-up	1	93	−0.06 [−0.46, 0.35]	NA
Long-term follow-up	0	–	–	–
**Stress-related outcomes (SMD)**				
All studies	9	876	0.02 [−0.11, 0.15]	0 %, *p* = 0.86
RCT	4	288	−0.06 [−0.29, 0.18]	0 %, *p* = 0.57
NRCT	4	563	0.04 [−0.13, 0.21]	0 %, *p* = 0.74
Pre-Post	1	25	0.18 [−0.38, 0.73]	NA
Placebo	4	291	0.00 [−0.23, 0.23]	0 %, *p* = 0.83
Non-placebo	5	608	0.02 [−0.14, 0.18]	0 %, *p* = 0.59
Short-term follow-up	5	576	0.06 [−0.10, 0.22]	0 %, *p* = 0.78
Medium-term follow-up	6	424	−0.05 [−0.24, 0.15]	0 %, *p* = 0.74
Long-term follow-up	1	65	0.04 [−0.46, 0.53]	NA
**Job/life satisfaction (SMD)**				
All studies	10	1935	*0.26 [0.05, 0.46]	78 %, *p* = 0.001
RCT	3	963	0.04 [−0.17, 0.25]	52 %, *p* = 0.12
NRCT	3	456	0.59 [−0.18, 1.36]	91 %, *p* = 0.001
Pre-Post	4	488	*0.22 [0.08, 0.37]	0 %, *p* = 0.50
Placebo	3	876	0.14 [0.00, 0.28]	0 %, *p* = 0.79
Non-placebo	3	639	0.47 [−0.29, 1.23]	95 %, *p* = 0.001
Short-term follow-up	5	862	0.33 [−0.17, 0.84]	91 %, *p* = 0.001
Medium-term follow-up	5	1175	*0.20 [0.09, 0.31]	0 %, *p* = 0.68
Long-term follow-up	1	65	0.18 [−0.32, 0.68]	NA
**Other satisfaction (SMD)**				
All studies	4	494	0.10 [−0.26, 0.46]	75 %, *p* = 0.008
RCT	0	–	–	–
NRCT	2	226	−0.12 [−1.33, 1.09]	91 %, *p* = 0.001
Pre-Post	2	268	0.14 [−0.08, 0.35]	0 %, *p* = 0.40
Placebo	0	–	–	–
Non-placebo	2	226	−0.12 [−1.33, 1.09]	91 %, *p* = 0.001
Short-term follow-up	3	441	−0.03 [−0.44, 0.39]	74 %, *p* = 0.02
Medium-term follow-up	2	238	*0.39 [0.16, 0.62]	0 %, *p* = 0.44
Long-term follow-up	0	–	–	–
**Staff attitude (SMD)**				
All studies	2	254	*0.23 [0.05, 0.45]	0 %, *p* = 0.35
RCT	0	–	–	–
NRCT	1	65	0.02 [−0.48, 0.52]	NA
Pre-Post	1	180	*0.28 [0.05, 0.52]	NA
Placebo	1	65	0.02 [−0.48, 0.52]	NA
Non-placebo	0	–	–	–
Short-term follow-up	0	–	–	–
Medium-term follow-up	2	293	*0.31 [0.11, 0.52]	0 %, *p* = 0.58
Long-term follow-up	1	65	0.02 [−0.29, 0.33]	NA
**Self-esteem (SMD)**				
All studies	2	141	0.14 [−0.50, 0.79]	65 %, *p* = 0.09
RCT	0	–	–	–
NRCT	2	141	0.14 [−0.50, 0.79]	65 %, *p* = 0.09
Pre-Post	0	–	–	–
Placebo	0	–	–	–
Non-placebo	2	141	0.14 [−0.50, 0.79]	65 %, *p* = 0.09
Short-term follow-up	2	141	0.14 [−0.50, 0.79]	65 %, *p* = 0.09
Medium-term follow-up	1	41	0.53 [−0.14, 1.20]	NA
Long-term follow-up	0	–	–	–

##### 3.1.1.1 Staff turnover rates

Two studies were included: one rated high risk of bias and the other rated very high risk of bias (Table 1; study details can be found in Supplementary file: Table 5). The odds of staff turnover were 53 % lower in the intervention group and were statistically significant. With a control probability of turnover of 34 %, such an odds ratio corresponds to a relative risk of 0.57, i.e., 43 % less risk of staff turnover in the intervention group. However, this result was largely based on a very high risk of bias study using a pre-post design.

##### 3.1.1.2 Absenteeism

Both included studies were RCTs (Table 1; study details can be found Supplementary file: Table 5). They were rated high and very high risk of bias. The standardised mean difference for staff absenteeism favoured the intervention group, but the result was not statistically significant.

##### 3.1.1.3 Stress-related outcomes

Nine studies measured staff stress-related outcomes (Table 1; study details can be found Supplementary file: Table 5), rated low to very high risk of bias. The standardised mean difference for stress-related outcomes marginally favoured the control group/no intervention, but the result was not statistically significant.

Stratified by study type, the standardised mean difference in RCT studies marginally favoured the intervention, whereas nRCT and Pre-Post studies marginally favoured the control group. There were no statistically significant differences between the study design subgroups.

Stratified by comparator type, the standardised mean difference for both placebo and non-place control trials suggested no intervention effect; and there were no statistically significant differences between the comparator type subgroups.

Subgroup analysis by follow-up duration included two subgroups: short-term and medium term. At short-term follow-up the standardised mean difference marginally favoured the control group. However, at medium-term follow-up the standardised mean difference marginally favoured the intervention. There were no significant differences between the follow-up duration subgroups.

##### 3.1.1.4 Job/life satisfaction

Ten studies (Table 1; study details can be found Supplementary file: Table 5) were included and were rated moderate to very high risk of bias. The standardised mean difference favoured the intervention and the result was statistically significant, albeit with statistically significant heterogeneity. After excluding Jung et al. (2020), the result was still statistically significant with no heterogeneity present.

Stratified by study type, the standardised mean difference in all study types favoured the intervention group. However, the result was statistically significant only for the Pre-Post subgroup, for which heterogeneity test was not statistically significant.

Stratified by comparator type, the standardised mean difference in controlled trials using placebo marginally favoured the intervention group. In the non-placebo that difference was more pronounced. However, in both cases, these results were not statistically significant. There was a statistically significant difference between results reported by studies using placebo versus non-placebo control groups (*I*^2^ = 88 %, *p* < 0.00001). The largest contribution to the high heterogeneity was from Jung et al. (2020), a non-placebo study, exclusion of which made the heterogeneity test statistically non-significant (*I*^2^ = 30 %, *p* = 0.22).

Subgroup analysis by follow-up duration included two subgroups: short-term and medium term. At short-term follow-up the standardised mean difference favoured the intervention group. Jung et al. (2020) study results were the largest contributor to the high heterogeneity for the short-term follow- up subgroup (*I*^2^ = 88 %, *p* < 0.00001). At medium-term follow-up the standardised mean difference favoured the intervention group and it was statistically significant with no heterogeneity.

##### 3.1.1.5 Other satisfaction

Four studies measured staff's other satisfaction outcomes, such as intrinsic satisfaction or satisfaction with nursing care (Table 1; study details can be found Supplementary file: Table 5). They were rated high to very high risk of bias. The standardised mean difference for stress-related outcomes favoured the intervention. Notably, the largest contributor to the high heterogeneity for this comparison was the Mackenzie and Peragine (2003) study (rated high risk of bias) which reported results favouring the control group. Excluding this study resulted in achieving statistical significance for this comparison.

Stratified by study type and comparator type, the standardised mean difference for other satisfaction outcomes in two non-placebo nRCT studies favoured the control group. In Pre-Post studies, the standardised mean difference favoured the intervention group. There were no statistically significant differences.

Subgroup analysis by follow-up duration included two subgroups: short-term and medium term. At short-term follow-up the standardised mean difference marginally favoured no intervention. At medium-term follow-up, the standardised mean difference favoured the intervention. This result was statistically significant. Overall, the difference between follow-up duration subgroups was approaching statistical significance (*p* = 0.09).

##### 3.1.1.6 Staff attitude (towards dementia)

Two studies measuring staff's attitude towards dementia were included (Table 1; study details can be found Supplementary file: Table 5) and rated high and very high risk of bias. The standardised mean difference favoured the intervention group and the result was statistically significant. However, this result was largely based on a study with a very high risk of bias using a pre-post design (Inker et al., 2021).

##### 3.1.1.7 Self-esteem

Two studies were included (Table 1; study details can be found Supplementary file: Table 5) and rated high and very high risk of bias. The standardised mean difference favoured the intervention group, but it was not statistically significant.

### 3.2 Interpersonal skills-based interventions

Twelve studies evaluated interpersonal skills-based interventions (mean study sample size was *n* = 161, IQR = 116; Supplementary file: Table 5). Three studies were rated ‘moderate risk of bias’. The remaining studies were rated ‘high’ (*n* = 7) or ‘very high risk of bias’ (*n* = 2; Supplementary file: Table 5). The most common issues affecting the risk of bias in the included studies were lack of randomisation and baseline differences between study groups.

Five studies focused exclusively on support workers. Other studies either included other health staff groups (for example, registered nurses; *n* = 5) or did not provide enough information to determine what the participants’ actual work roles were (for example, “study included professional caregivers”; *n* = 2). Five studies used an RCT design, five studies used a nRCT design, and two used a Pre-Post design. Six studies were conducted in the USA, three in the Netherlands, two in Germany, and one in Australia. The most common setting was nursing homes (*n* = 9); one study included long-term care facilities, one homes for elderly, and one assisted living facilities. Intervention durations varied between one day and nine months, with a median of two weeks. Four studies did not report intervention duration.

In seven studies, the core intervention component was interpersonal skills training. Three studies used a mix of interpersonal skills training and education sessions; one study used interpersonal skills training and mentoring; one study used interpersonal skills training, education sessions and mentoring. Ten studies tested interventions focusing on improving staff's interpersonal skills with people with dementia. One study looked at improving interpersonal skills with a focus on person-centredness, and one study focused on interpersonal skills in interactions with residents’ families. Seven studies reported testing interventions underpinned by theory, e.g., Person-Centred Care, Communication Enhancement Model, and Social Learning Theory. Five studies did not report use of a theoretical model. Apart from two studies testing an original intervention and its adaptation, all studies tested unique interventions.

#### 3.2.1 Interpersonal skills-based interventions meta-analyses

The included studies evaluated seven psychosocial and turnover-related outcomes. However, due to no data being available for meta-analysis or less than two studies in the comparison group, only three types of outcomes were analysed (Table 2) and are reported below.

**Table 2:** Summary of effect sizes for each outcome comparison for interpersonal skills-based interventions (statistically significant effect sizes are bolded and marked with ‘*’).

Outcome	Number of studies	Number of participants	Odds ratio or SMD	*I*^2^ (heterogeneity)
**Turnover (OR)**				
All studies	2	192	0.48 [0.05, 4.23]	79 %, *p* = 0.03
RCT	1	88	*0.15 [0.03, 0.71]	NA
NRCT	0	–	–	–
Pre-Post	1	104	1.38 [0.41, 4.69]	NA
Placebo	0	–	–	–
Non-placebo	1	88	*0.15 [0.03, 0.71]	NA
Short-term follow-up	1	104	1.38 [0.41, 4.69]	NA
Medium-term follow-up	1	88	*0.15 [0.03, 0.71]	NA
Long-term follow-up	0	–	–	–
**Stress-related outcomes (SMD)**				
All studies	2	51	−0.12 [−0.68, 0.45]	30 %, *p* = 0.23
RCT	0	–	–	–
NRCT	1	26	−0.49 [−1.29, 0.32]	NA
Pre-Post	1	25	0.11 [−0.45, 0.66]	NA
Placebo	0	–	–	–
Non-placebo	1	26	−0.49 [−1.29, 0.32]	NA
Short-term follow-up	2	51	−0.12 [−0.68, 0.45]	30 %, *p* = 0.23
Medium-term follow-up	0	–	–	–
Long-term follow-up	0	–	–	–
**Job/life satisfaction (SMD)**				
All studies	3	188	0.28 [−0.11, 0.66]	42 %, *p* = 0.18
RCT	0	–	–	–
NRCT	2	163	0.31 [−0.30, 0.92]	65 %, *p* = 0.09
Pre-Post	1	25	0.14 [−0.42, 0.70]	NA
Placebo	0	–	–	–
Non-placebo	2	163	0.31 [−0.30, 0.92]	65 %, *p* = 0.09
Short-term follow-up	2	77	0.05 [−0.35, 0.46]	0 %, *p* = 0.65
Medium-term follow-up	1	111	*0.58 [0.17, 0.98]	NA
Long-term follow-up	0	–	–	–

##### 3.2.1.1 Turnover rates

Two studies were included (Table 2; study details can be found Supplementary file: Table 5) and rated high and very high risk of bias. The odds of staff turnover were 52 % lower in the intervention group. With a control probability of turnover of 12 %, such an odds ratio corresponds to a relative risk of 0.51, i.e., 49 % less risk of staff turnover in the intervention group.

##### 3.2.1.2 Stress-related outcomes

Two studies were included (Table 2; study details can be found Supplementary file: Table 5) and rated high and very high risk of bias. The standardised mean difference favoured the intervention group, however, the result was not statistically significant.

##### 3.2.1.3 Job/life satisfaction

Three studies were included (Table 2; study details can be found Supplementary file: Table 5) and rated high to very high risk of bias. The standardised mean difference favoured the intervention group, but the result was not statistically significant.

Stratified by study type, the standardised mean difference in non-placebo nRCT studies favoured the intervention group. The Pre-Post subgroup included only one study. The two groups were not significantly different.

Subgroup analysis by follow-up duration included one subgroup: short-term follow-up. At short-term follow-up the standardised mean difference marginally favoured the intervention, but was not statistically significant.

### 3.3 Team-building interventions

Nine studies evaluated team building interventions (mean study sample size was *n* = 223, IQR = 208; *n* = 2 studies did not report the sample size; Supplementary file: Table 5). One study was rated ‘moderate risk of bias’. The remaining studies were rated ‘high’ (*n* = 5) or ‘very high risk of bias’ (*n* = 3; Supplementary file: Table 5). The most common issues affecting risk of bias in the included studies were lack of randomisation and baseline differences between study groups.

Six studies focused exclusively on support workers. Three studies included other health staff groups (for example, registered nurses). Two studies used an RCT design, four studies used a nRCT design, and three used a Pre-Post design. Eight studies were conducted in the USA and one in Sweden. The most common setting was nursing homes (*n* = 6); two studies included long-term care facilities, and one a mix of nursing homes and in-home care. Intervention duration varied between one and 18 months, with a median of six months.

The included studies used a range of intervention components to address the overarching goal of improving teamwork and team dynamics. In four studies the core intervention component were team meetings; two studies used a mix of mentoring, education sessions and interpersonal skills training; two studies used a mix of mentoring and education sessions; and one study used rewards. Three studies reported their interventions to be underpinned by empowerment-based theories (for example, Zimmerman's Empowerment Theory); two studies reported their interventions to be based on peer-mentoring concepts; one on the Participative Decision Making Theory, and three did not specify any underpinning theories. Apart from two studies testing the same intervention and reported in the same paper, all studies tested new interventions.

#### 3.3.1 Team-building interventions meta-analyses

The included studies evaluated twelve psychosocial and turnover-related outcomes. However, due to no data being available for meta-analysis or fewer than two studies in the comparison group, only five types of outcomes were analysed (Table 3) and are reported below.

**Table 3:** Summary of effect sizes for each outcome comparison for team-building interventions (statistically significant effect sizes are bolded and marked with ‘*’).

Outcome	Number of studies	Number of participants	Odds ratio or SMD	*I*^2^ (heterogeneity)
**Turnover (OR)**				
All studies	5	8604	0.65 [0.33, 1.28]	79 %, *p* = 0.0008
RCT	2	7684	1.09 [0.69, 1.71]	0 %, *p* = 0.35
NRCT	3	920	0.45 [0.16, 1.25]	82 %, *p* = 0.004
Pre-Post	0	–	**–**	–
Placebo	0	–	**–**	–
Non-placebo	5	8604	0.65 [0.33, 1.28]	79 %, *p* = 0.0008
Short-term follow-up	1	32	3.21 [0.32, 32.60]	NA
Medium-term follow-up	3	925	0.55 [0.16, 1.97]	87 %, *p* = 0.0005
Long-term follow-up	1	757	*0.56 [0.42, 0.75]	NA
**Intention to quit (SMD)**				
All studies	2	132	−0.32 [−0.82, 0.18]	32 %, *p* = 0.22
RCT	0	–	–	–
NRCT	2	132	−0.32 [−0.82, 0.18]	32 %, *p* = 0.22
Pre-Post	0	–	–	–
Placebo	0	–	–	–
Non-placebo	2	132	−0.32 [−0.82, 0.18]	32 %, *p* = 0.22
Short-term follow-up	0	–	–	–
Medium-term follow-up	1	25	−0.73 [−1.56, 0.10]	NA
Long-term follow-up	1	107	−0.16 [−0.54, 0.22]	NA
**Stress-related outcomes (SMD)**				
All studies	2	145	−0.08 [−0.52, 0.37]	40 %, *p* = 0.20
RCT	1	46	−0.36 [−0.94, 0.22]	NA
NRCT	1	99	0.11 [−0.29, 0.51]	NA
Pre-Post	0	–	–	–
Placebo	0	–	–	–
Non-placebo	2	145	−0.08 [−0.52, 0.37]	40 %, *p* = 0.20
Short-term follow-up	1	46	−0.36 [−0.94, 0.22]	NA
Medium-term follow-up	0	–	**–**	–
Long-term follow-up	1	99	0.11 [−0.29, 0.51]	NA
**Job/life satisfaction (SMD)**				
All studies	2	193	−0.07 [−0.50, 0.37]	56 %, *p* = 0.13
RCT	0	–	–	–
NRCT	2	193	−0.07 [−0.50, 0.37]	56 %, *p* = 0.13
Pre-Post	0	–	–	–
Placebo	0	–	–	–
Non-placebo	2	193	−0.07 [−0.50, 0.37]	56 %, *p* = 0.13
Short-term follow-up	0	–	–	–
Medium-term follow-up	1	81	−0.30 [−0.75, 0.14]	NA
Long-term follow-up	1	112	0.14 [−0.23, 0.52]	NA
**Self-esteem (SMD)**				
All studies	3	232	−0.08 [−0.34, 0.18]	0 %, *p* = 0.42
RCT	1	46	0.26 [−0.32, 0.84]	NA
NRCT	2	186	−0.16 [−0.45, 0.13]	0 %, *p* = 0.81
Pre-Post	0	–	–	–
Placebo	0	–	–	–
Non-placebo	3	232	−0.08 [−0.34, 0.18]	0 %, *p* = 0.42
Short-term follow-up	1	46	0.26 [−0.32, 0.84]	NA
Medium-term follow-up	1	80	−0.12 [−0.56, 0.32]	NA
Long-term follow-up	1	106	−0.20 [−0.58, 0.19]	NA

##### 3.3.1.1 Turnover rates

Five studies were included (Table 3; study details can be found Supplementary file: Table 5) and were rated moderate to high risk of bias. The odds of staff turnover were 35 % lower in the intervention group. With a control probability of turnover of 32 %, such an odds ratio corresponds to a relative risk of 0.73, i.e. 27 % less risk of staff turnover in the intervention group. The largest contributor to high heterogeneity in comparisons for this outcome was Webb (2003) study (high risk of bias) which reported results favouring the control group. Excluding this study did not result in achieving statistical significance in any of the comparisons.

Stratified by study type, the odds of staff turnover were 9 % higher in the intervention group in RCT studies. With a control probability of turnover of 10 %, such an odds ratio corresponds to a relative risk of 1.08, i.e. 8 % more risk of staff turnover in the intervention group. In nRCT studies, the odds of staff turnover were 55 % lower in the intervention group. With a control probability of turnover of 54 %, such an odds ratio corresponds to a relative risk of 0.64, i.e., 36 % less risk of staff turnover in the intervention group. The two groups were not significantly different.

All five studies used non-placebo control groups, so no additional analysis by comparator type was necessary.

Subgroup analysis by follow-up duration included one subgroup: medium-term follow-up. The odds of turnover were 45 % lower in the intervention group. With a control probability of turnover of 20 %, such an odds ratio corresponds to a relative risk of 0.61, i.e., 39 % less risk of staff turnover in the intervention group.

##### 3.3.1.2 Intention to quit

Two nRCT studies were included (Table 3; study details can be found Supplementary file: Table 5) and rated moderate and high risk of bias. The standardised mean difference favoured the intervention group, but was not statistically significant.

##### 3.3.1.3 Stress-related outcomes

Two studies were included and (Table 3; study details can be found Supplementary file: Table 5) rated high risk of bias. The standardised mean difference favoured the intervention group, but was not statistically significant.

##### 3.3.1.4 Job/life satisfaction

Two studies were included (Table 3; study details can be found Supplementary file: Table 5) and rated high risk of bias. The standardised mean difference for job/life satisfaction favoured the control group, but was not statistically significant.

##### 3.3.1.5 Self-esteem

Three studies were included (Table 3; study details can be found Supplementary file: Table 5) and rated high risk of bias. The standardised mean difference favoured the control group, but was not statistically significant. All three studies used non-placebo control groups, so no additional analysis by comparator type was warranted.

In nRCT studies, the standardised mean difference marginally favoured no intervention, but was not statistically significant.

Subgroup analysis by follow-up duration included one subgroup – medium-term follow-up. The standardised mean difference marginally favoured no intervention; not statistically significant.

### 3.4 Self-care interventions

Four studies evaluated self-care interventions (mean study sample size was *n* = 58, IQR = 58; Supplementary file: Table 5). One study was rated ‘moderate risk of bias’. The remaining studies were rated ‘high’ (*n* = 1) or ‘very high risk of bias’ (*n* = 2; Supplementary file: Table 5). The most common issues affecting risk of bias in the included studies were lack of blinding, concealment and randomisation, and differences between study groups at baseline.

Three studies focused exclusively on support workers; one study also included registered nurses. One study used an RCT design, one used a nRCT design, and two used a Pre-Post design. Three studies were conducted in the USA and one in Norway. Two studies were conducted in nursing homes, and another two at long-term care facilities. Intervention durations were: one day, eight weeks, and three and six months.

The included studies used a range of intervention components to address the overarching goal of getting support workers to practice self-care. In one study the core intervention component were weekly exercise sessions; one study used a mix of education sessions and relaxation practice; one study used a mix of education sessions and self-care practices, and one study used a mix of education sessions, mentoring, exercise sessions, and nutritional advice. One study reported its intervention to be underpinned by the Aerobic Fitness Model; one study based their interventions on the Socioecological model; one focused on the concept of compassion fatigue, and one on the concept of mindfulness. All studies tested new interventions.

#### 3.4.1 Self-care interventions meta-analyses

The included studies evaluated three psychosocial outcomes and are reported below (Table 4).

**Table 4:** Summary of effect sizes for each outcome comparison for self-care interventions (statistically significant effect sizes are bolded and marked with ‘*’).

Outcome	Number of studies	Number of participants	Odds ratio or SMD	*I*^2^ (heterogeneity)
**Stress-related outcomes (SMD)**				
All studies	3	103	−0.25 [−0.57, 0.08]	0 %, *p* = 0.58
RCT	0	–	–	–
NRCT	1	39	−0.00 [−0.65, 0.65]	NA
Pre-Post	2	64	−0.33 [−0.71, 0.05]	0 %, *p* = 0.55
Placebo	1	39	−0.00 [−0.65, 0.65]	NA
Non-placebo	0	–	–	–
Short-term follow-up	3	103	−1.18 [−2.91, 0.54]	0 %, *p* = 0.37
Medium-term follow-up	1	39	−0.00 [−0.65, 0.65]	NA
Long-term follow-up		–	–	–
**Job/life satisfaction (SMD)**				
All studies	2	124	−0.14 [−0.50, 0.22]	0 %, *p* = 0.52
RCT	1	85	−0.22 [−0.65, 0.21]	NA
NRCT	1	39	0.04 [−0.61, 0.68]	NA
Pre-Post	0	–	–	–
Placebo	1	39	0.04 [−0.61, 0.68]	NA
Non-placebo	1	85	−0.22 [−0.65, 0.21]	NA
Short-term follow-up	1	39	0.19 [−0.46, 0.84]	NA
Medium-term follow-up	2	124	−0.14 [−0.50, 0.22]	0 %, *p* = 0.52
Long-term follow-up	0	–	–	–
**Other satisfaction (SMD)**				
All studies	2	54	0.02 [−0.36, 0.39]	0 %, *p* = 0.88
RCT	0	–	–	–
NRCT	0	–	–	–
Pre-Post	2	54	0.02 [−0.36, 0.39]	0 %, *p* = 0.88
Placebo	0	–	–	–
Non-placebo	0	–	–	–
Short-term follow-up	2	54	0.02 [−0.36, 0.39]	0 %, *p* = 0.88
Medium-term follow-up	0	–	–	–
Long-term follow-up	0	–	–	–

##### 3.4.1.1 Stress-related outcomes

Three studies were included (Table 4; study details can be found Supplementary file: Table 5) and rated high and very high risk of bias. The standardised mean difference favoured the intervention group; however, the result was not statistically significant. Subgroup analysis by follow-up duration included one subgroup – short-term follow-up. The standardised mean difference favoured the intervention group but was not statistically significant.

##### 3.4.1.2 Job/life satisfaction

Two studies were included (Table 4; study details can be found Supplementary file: Table 5) and rated moderate and high risk of bias. The standardised mean difference favoured the control group, but it was not statistically significant.

##### 3.4.1.3 Other satisfaction (compassion satisfaction)

Two studies measuring staff's compassion satisfaction were included (Table 4; study details can be found Supplementary file: Table 5) and rated very high risk of bias. The standardised mean difference favoured the intervention group, but it was not statistically significant.

### 3.5 Summary of the meta-analysis findings

Most of the comparisons showed positive changes; however, the effect sizes were small or very small. Three of these comparisons were statistically significant: staff turnover (OR 0.47, 95 %CI: 0.37, 060), job/life satisfaction (SMD 0.26, 95 % CI: 0.05, 0.46) and staff attitude (specifically, staff attitude towards dementia; SMD 0.23, 95 % CI: 0.05, 0.45). Subgroup analyses by follow-up duration found that for many several outcome types (e.g., turnover and job/life satisfaction) in knowledge and interpersonal skills interventions the medium-term follow-up (3–12 months) results were statistically significant. Table 5 presents the summary of meta-analysis findings.

**Table 5:** Summary of main meta-analysis findings.

Outcome	Knowledge	Interpersonal	Team building	Self-care
Turnover (OR)	+ (*0.47)	+ (0.48)	+ (0.65)	NA
Intention to quit (SMD)	NA	NA	+ (−0.32)	NA
Absenteeism (SMD)	+ (−0.36)	NA	NA	NA
Stress (SMD)	0 (0.02)	0 (−0.12)	0 (−0.08)	+ (0.25)
Job/life satisfaction (SMD)	+ (*0.26)	+ (0.28)	0 (−0.07)	0 (−0.14)
Other satisfaction (SMD)	0 (0.1)	NA	NA	0 (0.02)
Staff attitude (SDM)	+ (*0.23)	NA	NA	NA
Self-esteem (SMD)	0 (0.14)	NA	0 (−0.08)	NA
‘0′ no effect or very small; ‘+’ positive small effect size; ‘*’- statistically significant				

## 4 Discussion

In this paper we synthesised findings from studies evaluating the evidence on effectiveness of interventions for improving psychosocial and turnover-related outcomes for support workers in aged care. This review has highlighted that while there are interventions that lead to improvements in the key outcomes, the evidence is of low certainty due to a range of limitations relating to the design and substantial heterogeneity of the included studies.

The included studies represented a broad range of approaches, from interventions focusing on teaching staff about symptoms of dementia (Zimmerman et al., 2010) to studies trialling exercise classes for support workers (Brox and Froøystein, 2005). Knowledge-based interventions were the most evaluated intervention type and reported by their authors to be particularly effective in improving stress-related and job/life/other satisfaction outcomes. Interpersonal skills-based interventions were also reported to be particularly effective in improving stress-related outcomes. Team-building interventions were reported to be especially effective in improving staff turnover and reducing intention to quit. Only one of the four self-care interventions reported positive changes in their target outcomes (stress and burnout), while the remaining three found no change.

Overall, the meta-analysis showed that interventions for aged care support workers were commonly associated with positive, albeit small and mostly non-significant, changes in work-related psychosocial outcomes (such as job/life satisfaction, stress, intention to quit) and turnover rates. With high between-study heterogeneity (intervention- and outcome-wise), scarce high-quality evidence and most of the meta-analyses not yielding any statistically significant results, the evidence provides limited certainty in relation to the effectiveness of the identified interventions. The evidence for effectiveness of knowledge-based interventions appears the most convincing, with statistically significant improvements reported for turnover rates, job/life satisfaction and staff attitude.

One of the key findings of this review is that the effectiveness of any specific approaches varies between settings. This review found that similar interventions (e.g., knowledge-based interventions focusing on understanding dementia) may lead to contradictory results (e.g., studies by Cheng (2008) and Zimmerman et al. (2010), see Supplementary file: Table 5) or no effects (da Silva Serelli et al., 2017) when used in different countries/contexts (Taiwan vs USA vs Brazil). One possible explanation for this is that these contrasting effects may result from between-study differences in the intervention fidelity, adherence, the outcome measure used, cultural differences or a combination of these. Importantly, most of the included studies did not report any findings relating to intervention fidelity, and many used ad hoc or unvalidated outcome measures. Using validated measures and reporting intervention fidelity are crucial to accurate interpretation of study findings (Del Greco, Walop, and McCarthy, 1987; McGee et al., 2018). Another plausible explanation for observing contradictory or no effects is that the needs and preferences of these studies’ participants were diverse, therefore requiring an adaptation of the existing approaches. Thus, we recommend that any existing and new interventions be tailored to the specific implementation context and the support worker's personal needs and preferences, for example, relating to their cultural background (Bird et al., 1996; Gallagher‐Thompson et al., 2003; Van Duyn et al., 2007).

The results of subgroup meta-analysis by follow-up duration indicated the effects of the interventions were more pronounced when follow-up scores were collected between three and 12 months post-implementation. This was perhaps unsurprising, given that the majority of the studies tested interventions lasting two or more months. It appears that interventions aiming to improve psychosocial and turnover-related outcomes may need to last longer than three months. However, interventions with long duration may lead to lower adherence and higher drop-out rates (Younge et al., 2015), which can be impacted further by high staff turnover reported for support workers (Jorgensen et al., 2009). We recommend a careful consideration of the above when developing or implementing an intervention for aged care support workers.

### 4.1 Caveats and limitations

There are a number of caveats and limitations to consider when interpreting the findings of this work. First, the literature search and data extraction were primarily conducted by only one author (Karol J. Czuba), and this may have resulted in an unconsciously biased selection or omission of papers. However, the selection criteria were developed and reviewed by all authors, and any ambiguities were resolved by discussion with the co-authors. Additionally, a subsample of *n* = 50 abstracts were reviewed by Frances Czuba. Second, we did not exclude studies based on their methodological quality. While including such studies may provide additional evidence on potential causal inferences, the increased risk of bias this inclusion carries requires caution in interpreting and generalising the review findings. Third, to manage the high intervention heterogeneity, we arbitrarily grouped the studies by intervention type. A different grouping might have produced different results. Furthermore, despite the relatively large number of included studies, due to high heterogeneity, the number of studies in many subgroups was still small. However, given the scarcity of high-quality studies and the mostly very small or small effect sizes, the overall conclusion of this review would likely remain the same even if a different grouping approach was used. Finally, the included studies used over 50 different outcome measures, with many of them not psychometrically tested or validated. This meant that many studies could not be included in the meta-analyses, or their inclusion resulted in increased heterogeneity, making the interpretation of results even more challenging.

## 5 Conclusion

This study found that the published studies on interventions for aged care support workers were generally associated with positive, albeit small and mostly non-significant, changes in work-related psychosocial outcomes (such as job/life satisfaction, stress, intention to quit) and turnover rates. The evidence for effectiveness of knowledge-based interventions appears the most convincing, with statistically significant improvements reported for turnover rates, job/life satisfaction and staff attitude. However, the effect of interventions appears to vary depending on the specific setting. More high-quality studies testing such interventions are needed.

## Funding

This work was supported by the 10.13039/501100001505Health Research Council of New Zealand (grant number: 17/018) and Postgraduate Research funds offered to all doctoral students enrolled in the Faculty of Health and Environmental Sciences at Auckland University of Technology.

## Declaration of Competing Interest

The authors declare that they have no known competing financial interests or personal relationships that could have appeared to influence the work reported in this paper.

## Data Availability

Extracted data are available upon request to the corresponding author.

## Appendix Supplementary materials

Image, application 1

## Footnotes

Supplementary material associated with this article can be found, in the online version, at doi:10.1016/j.ijnsa.2023.100158.
