# Supplementary material for: The effectiveness of peer-led interventions to improve work-related psychosocial outcomes and reduce turnover of support workers in residential aged care: A systematic review and meta-analysis
Source: Int J Nurs Stud Adv. 2023 Oct 15;5:100158. doi: 10.1016/j.ijnsa.2023.100158 (PMC11080450; doi:10.1016/j.ijnsa.2023.100158)
Supplement: Supplementary file 1 [file mmc1.docx]

**Supplementary file: “The effectiveness of interventions to improve work-related psychosocial outcomes and reduce turnover of support workers in residential aged care: a systematic review and meta-analysis.”**

# Appendix 1. Search strategy

| **Scopus** | ( TITLE-ABS-KEY ( "support worker*" OR "nursing assistant*" OR "healthcare assistant*" OR "carer*" OR "caregiver*" OR "nurse* aid*" OR "nursing aid*" OR "nurse* aide*" OR "nursing aide*" OR "therapy aide*" OR "therapy aid*" OR "therapy assistant*" OR "direct care staff*" OR "personal care staff*" OR "personal care assistant*" OR cna* OR "certified nursing assistant*" )  **AND**  TITLE-ABS-KEY ( retention* OR turnover* OR "mental health" OR "wellbeing" OR "well-being" OR "well being" OR stress OR burnout OR "burn out" OR burden OR satisfaction OR "quality of life" OR qol OR "role ambiguity" OR "role conflict" OR "intention to leave" OR "self-esteem" )  **AND**  TITLE-ABS-KEY ( "aged care facilit*" OR "rest home*" OR "private hospital*" OR "retirement village*" OR "care home*" OR "home for the aged" OR "nursing home" OR "long term care" OR "longterm care" OR "long-term care" OR "residential aged care" OR "residential care" OR "residential facility" OR "residential home" ) )  **AND**  ( LIMIT-TO ( LANGUAGE , "English " ) ) |
| --- | --- |
| **EBSCO** | AB "support worker*" OR AB "nursing assistant*" OR AB "healthcare assistant*" OR AB "carer*" OR AB "caregiver*" OR AB "nurs* aid*" OR AB "therapy aid*" OR AB "therapy assistant*" OR AB "direct care staff" or "personal care staff" or "personal care assistant*" OR AB cna* or "certified nurs* assistant*"  **AND**  AB retention* OR AB turnover* OR AB "mental health" OR AB wellbeing* or "well-being" or "well being" OR AB stress* OR AB burnout or "burn-out" or "burn out" OR AB burden OR AB satisfaction or "quality of life" or qol OR AB "role ambiguity" or "role conflict" or "intention to leave" or "self-esteem"  **AND**  AB "aged care facilit*" or "rest home*" or "private hospital*" OR AB "retirement village*" or "care home*" or "home for the aged" OR AB "nursing home" or "long term care" or "long-term care" or "longterm care" OR AB "residential aged care" or "residential care" or "residential facility" or "residential home" |
| **Pubmed** | "support worker*"[Title/Abstract] OR "nursing assistant*"[Title/Abstract] OR "healthcare assistant*"[Title/Abstract] OR "carer*"[Title/Abstract] OR "caregiver*"[Title/Abstract] OR "nurs* aid*"[Title/Abstract] OR "therapy aid*"[Title/Abstract] OR "therapy assistant*"[Title/Abstract] OR "direct care staff"[Title/Abstract] or "personal care staff"[Title/Abstract] or "personal care assistant*"[Title/Abstract] OR cna*[Title/Abstract] or "certified nurs* assistant*" [Title/Abstract]  **AND**  retention*[Title/Abstract] OR turnover*[Title/Abstract] OR "mental health"[Title/Abstract] OR wellbeing*[Title/Abstract] or "well-being"[Title/Abstract] or "well being"[Title/Abstract] OR stress*[Title/Abstract] OR burnout[Title/Abstract] or "burn-out"[Title/Abstract] or "burn out"[Title/Abstract] OR burden[Title/Abstract] OR satisfaction[Title/Abstract] or "quality of life"[Title/Abstract] or qol[Title/Abstract] OR "role ambiguity"[Title/Abstract] or "role conflict"[Title/Abstract] or "intention to leave"[Title/Abstract] or "self-esteem"[Title/Abstract]  **AND**  "aged care facilit*"[Title/Abstract] or "rest home*"[Title/Abstract] or "private hospital*"[Title/Abstract] OR "retirement village*"[Title/Abstract] or "care home*"[Title/Abstract] or "home for the aged"[Title/Abstract] OR "nursing home"[Title/Abstract] or "long term care"[Title/Abstract] or "long-term care"[Title/Abstract] or "longterm care"[Title/Abstract] OR "residential aged care"[Title/Abstract] or "residential care"[Title/Abstract] or "residential facility"[Title/Abstract] or "residential home"[Title/Abstract] |
| **British Journal of Healthcare Assistants** | retention* OR turnover* OR "mental health" OR wellbeing* or "well-being" or "well being" OR stress* OR burnout or "burn-out" or "burn out" OR burden OR satisfaction or "quality of life" or qol OR "role ambiguity" or "role conflict" or "intention to leave" or "self-esteem" |

# Appendix 2. Main intervention components

Six main intervention components were identified:

1. Education sessions and materials – provision of information on a specific topic related to aged residential care, e.g. teaching about dementia and its consequences, or person-centred care approach;

2. Interpersonal skills training – provision of interpersonal skills training, e.g. group session during which staff practiced a specific way of communicating with others.

3. Team meetings – provision of new meeting structures to facilitate staff’s discussions and/or brainstorming, e.g. adding a 30-minute debriefing session following each handover meeting.

4. Rewards – provision of incentives serving as rewards in recognition of good service.

5. Relaxation, exercise and diet – provision of structured exercise or meditations sessions, and/or nutritional advice.

6. Mentoring – training a support worker to become a mentor/trainer; could include elements of any of the above.

# Appendix 3. Full list of outcomes reported by the included studies.

Over 35 outcomes were identified in the included studies. These outcomes were classified into 13 types of outcomes:

1. Turnover-related – turnover, turnover rates, revolving door turnover, and similar;
2. Retention rates;
3. Intention to quit – intention to quit, intention to leave, job commitment, and similar.
4. Absenteeism – absenteeism, sick leave utilisation, and similar;
5. Stress-related – stress levels, burnout, burden, strain, workload, coping style, distress, and similar. Where Maslach Burnout^[[1]](#footnote-1)^ Inventory was used, we considered the Emotional Exhaustion total score;
6. General satisfaction – job satisfaction, life satisfaction, general satisfaction, happiness, and similar;
7. Other satisfaction – specific types of satisfaction, for example satisfaction with supervision;
8. Quality of life;
9. Self-esteem – self-esteem, self-efficacy, empowerment level, and similar;
10. General health – general health, health, well-being, and similar;
11. Depression – depression, anxiety, happiness, depressive symptomatology, and similar;
12. Staff attitude – staff attitude, staff morale, and similar;
13. Other – outcomes not fitting under any of the abovementioned categories.

# Appendix 4. Summary tables for each intervention type.

## Table 1. Knowledge-based interventions summary table.

| **Design** | **Intervention** | **Population** | **Psychosocial outcomes** | **Turnover-related outcomes** |
| --- | --- | --- | --- | --- |
| **Almquist, E. et al. (1981); Risk of bias: Very high;**  **Comment:** No control group. The study was conducted on a group of n=29 NAs and n=52 LPNs who underwent the training separately, and the results were reported separately for each group. Life satisfaction was measured with the Life Satisfaction Scale (Adams, 1969). | | | | |
| A before and after study design. Data was collected at baseline and six weeks later (immediately post-intervention). | The goal of this training was to provide NAs with knowledge about long term care and improve their attitudes toward elderly.  The course was delivered over a period of six weeks. Training sessions of about 90 minutes were held twice a week. The education focused on anatomy, physiology of ageing, physical and psychosocial problems of the elderly, attitudes, and communication skills.  It was not reported who delivered the training. | n=29 NAs in n=3 nursing homes in Florida, US. | **Life satisfaction**  No significant differences were found. | Not applicable |
| **Barbosa, A. et al.** **(2016); Risk of bias: Moderate;**  **Comment:** Concealment method not reported. Researchers were not blind to group allocation. Perceived stress was measured with the Portuguese version of the Perceived Stress Scale (Pais Ribeiro & Marques, 2009); burnout was measured with the Maslach Burnout Inventory Human Services Survey (Melo et al., 1999); job satisfaction was measured with the Minnesota Satisfaction Questionnaire (Ferreira at al., 2009). | | | | |
| A cluster-randomised controlled trial with outcomes measurements collected at baseline, and at two weeks and six months post-intervention. | The control group received the Person Centred Care-Based Education program.  The education component of the program provided NAs with: a) principles of integrating person centred care into practice, b) knowledge about dementia, c) a range of person centred care-based interaction strategies.  The experimental group received the same program with an addition of a supportive component (The Person-Centred Care-Based Psychoeducational).  The experimental group program aimed to provide NAs with coping strategies to manage work-related stress and prevent burnout. It included training in time management, assertiveness and problem solving. At the end of each session, relaxation techniques, stretching and strengthening exercises were practiced.  Both groups programs were coordinated by a gerontologist and a physical therapist and included eight weekly 90-minute sessions. Additionally, during the three days following the education sessions the two coordinators assisted the NAs during morning care and suggested ways of implementing a more person centred care approach. | n=53 NAs in n=4 residential aged care facilities in Portugal; n=24 in the experimental group and n=29 in the control group. | **Perceived stress**  Both groups reported significantly higher levels of stress at six-month follow-up than at baseline and two-week follow-up.  No other significant changes were found.  **Burnout**  A significant time interaction effect on the personal accomplishment subscale was found. The scores for both groups declined immediately post-intervention. However, in the experimental group the scores had then improved at the last follow-up. In the control group, the scores declined further.  No other significant changes were found.  **Job satisfaction**  No significant changes were found. | Not applicable |
| **Beck, I. et al.** **(2013); Risk of bias: High;**  **Comment:** Allocation was not random, and groups differed at baseline. General job satisfaction was measured with the Job Satisfaction Questionnaire (Ekvall, 2001); satisfaction with nursing care was measured with the Psychosocial Aspects of Job Satisfaction scale (Engstrom et al., 2006); strain was measured with Strain Dementia Care Scale (Edberg et al., 2013); stress of conscience was measured with the Stress of Conscience Questionnaire (Glasberg et al., 2006). | | | | |
| A before and after study with a control group. Outcome data were collected immediately before and after the intervention, and at six months post-intervention. | Applying a palliative care approach in residential care. Consisted of seven two-hour circle sessions and three six-hour workshops. A circle leader participated in a three-day workshop with two follow-up days. The circle leaders ran the circle sessions and workshops were for nurse assistants (NA). The leader’s role was to facilitate the circle sessions and workshops rather than act as experts. The sessions focused on discussing and reflecting on texts and practical tasks carried out prior to the meetings, e.g. reading about the topic or interviewing a resident. The circle session topics included palliative care philosophy, older people’s experiences, autonomy, relatives’ role and support, life before death, and being a staff and a fellow human. The workshops focused on discussing how to change the practice based on the circle sessions teachings. | NAs in residential aged care facilities in Sweden; n=88 NAs in the intervention group; n=137 NAs in the control group. | **General job satisfaction**  A statistically significant change over time (worse) in the intervention group. No statistically significant changes were found in the control groups.  **Satisfaction with nursing care**  Statistically significant changes in internal motivation (worse) and criticism (better) over time in the intervention group.  Statistically significant changes in cooperation, and internal and external motivation (better) over time in the control group.  **Strain**  A statistically significant change over time was found for the intervention group (an increase in mean values, followed by a decrease). Post-hoc analysis found a significant decrease in strain between post-intervention and six months. No statistically significant changes were found in the control groups.  **Stress of Conscience**  No Changes. | Not applicable |
| **Bright-Long, L. E. (1990); Risk of bias: Very high;**  **Comment:** A single group design. Research design characteristics very general and lack some important information (e.g. total number of participants). Study results lack clarity. Paper only reports survey results and only to some of the items; no statistical tests were applied. Burden was measured with an adapted version of the Zarit Burden Interview, not previously validated. | | | | |
| A before and after study design. Measures were taken at baseline, and four months later, after completing the intervention. | The intervention was designed to train NAs to understand the “who, what, where, why and how” of dementia.  The program was delivered by the study researcher, an assistant professor of psychiatry. The program was delivered in weekly half-hour sessions. Each session was attended by a maximum of n=7 NAs. | NAs in a nursing home in New York, US; exact number of participants not reported. | **Burden**  No statistical tests were applied. The scores appeared to have slightly improved following the intervention. | Not applicable. |
| **Cheng, W. (2008)**; **Risk of bias: Very high;**  **Comment:** Only n=6 participants were support workers (nurse aides (NA)). Job satisfaction was measured with the Minnesota Satisfaction Questionnaire (Weis et al., 1967); health perception with the SF-36 subscale General Health Perception (Ware & Sherbourne, 1992); quality of life with Chinese WHOQoL (Yao, 2002); all other measures were developed by the investigator for the current study. It appears that scoring on health perception scale is incorrect – reversed items are scored the same as non-reversed. Most of the mean scores at T0 and T1 scores do not add up to what authors report as the differences between those scores. | | | | |
| An evaluation study using pre- and post-intervention measures at four timepoints: before intervention, within a week post-intervention, and one and three months post-intervention. | The Chinese version of Dementia Education Program. The focus was on behavioural and psychological symptoms of dementia (BPSD). Three teaching modules delivered over three weeks (one three-hour module a week). Delivered during work time. Each participant was given a certificate of completion. The modules were titled: Overview on Dementia; Related Issues on Dementia Care; and BPSD and Dementia Care. The program was delivered by the study investigator. | n=90 nursing staff from 55 nursing homes in Taiwan; the outcomes data is reported for n=45 direct nursing staff, only n=6 were NAs | **Attitude**  No improvement within one week post-intervention.  Improved (p<0.01) at 1 and 3 months post-intervention.  **Perceived Self-Efficacy**  Improved (p<0.001) at all timepoints.  **Caregiving Stress**  Improved (p<0.01) within one week post-intervention and improved (p<0.001) at one and three months post-intervention.  **Job Satisfaction**  No improvements observed.  **Health Perception**  No improvement within one week post-intervention and one month post-intervention.  Improved (p<0.01) at 3 months post-intervention.  **Quality of life**  No improvements observed. | Not applicable |
| **Coogle, C. et al. (2006); Risk of bias: Very high;**  **Comment**: No control group. A pre/post evaluation of a state-wide training for support workers. Job satisfaction was measured with the Minnesota Satisfaction Questionnaire (Weiss et al., 1967); career commitment was measured with the Career Commitment Measure (Carson & Bedeian, 1994). Authors suggest that the time factor (9-12 months for post-test) needs to be considered and argue that the improvements in satisfaction can be reasonably attributed to the intervention. However, they believe that the decline in career commitment could potentially be a result of the passage of time. | | | | |
| A single group before and after study. Data was collected at baseline, and at 9-12 months later. | This was a dementia care training program consisting of two phases.  Phase one: focused on person-centred care training. The program curriculum to deliver this 12-hour training, included: a) information on dementia, b) care need of people with dementia, c) treatments for people with dementia, d) environmental issues, e) behavioural management, and f) successful caregiver interventions. This phase was delivered by a range of trainers, including nurses, social workers, occupational therapists, and other. These trainers were prepared during a course of six eight-hour sessions on training staff.  Phase Two: utilised a train-the-trainer approach. NAs who had participated in the first phase were recruited to become trainers. They were then expected to teach their peers about care for people with dementia. The training focused on person-centred care and communication, behavioural management, and stress management. No information on duration of the second phase training was provided. | n=53 nursing staff (>80% in long term care facilities) in Virginia, US; 73.8% were CNAs. | **Extrinsic job satisfaction**  A significant improvement in extrinsic job satisfaction subscale.  **Intrinsic and total job satisfaction**  No significant differences were found. | **Career commitment**  A significant decline in total career commitment score, as well as in career identity subscale and career planning subscale.  No significant changes were found for career resilience subscale. |
| **Da Silva, L. et al.** **(2017); Risk of bias: Very high;**  **Comment:** No control group. Burden was measured with Zarit Burden Scale (Zarit et al., 1986); depression and anxiety were measured with the Beck Depression and Anxiety Inventory (Cunha, 2001); quality of life was measured with the Portuguese SF-36 (Ciconelli et al.,1999). | | | | |
| A single group before and after study design. Outcome data was collected at baseline, and 12 weeks later (6 weeks post-intervention). | The Staff Training for Assisted Living Residences (STAR) aimed to reduce the behavioural and psychological symptoms of dementia and caregiver burden. It consisted of 12 modules which included lectures, discussions, games, video vignettes, and handouts. It features information on dementia, communication skills, and introduction of ‘pleasant events’. It was delivered as a mixture of workshops (two x 2 hours each) and individual training sessions (four) over a six-week period.  The program was led by the study researcher – an experienced occupational therapist specialised in gerontology and dementia care training. | n=25 nursing staff (caregivers and nurses) in n=2 long term care facilities for people with dementia in Brazil. Exact number of caregivers and nurses not reported. | **Burden**  No significant differences were found.  **Depression**  No significant differences were found.  **Anxiety**  No significant differences were found.  **Quality of life**  No significant differences were found. | Not applicable. |
| **Dichter, M., et al. (2017); Risk of bias: High;**  **Comment:** No randomisation. Groups were not similar. Job satisfaction was measured using Copenhagen Psychosocial Questionnaire (Kristensen et al., 2005); staff attitudes were measured using the Approaches to Dementia Questionnaire (Lintern et al., 2000); caregiver burden was measured using the Copenhagen Burnout Inventory (Borritz et Kristensen, 2001). | | | | |
| A non-randomised controlled trial with three study arms. Data was collected at baseline, six and 18 months later.  Group A was familiar with the intervention prior to implementation. Group B was new to intervention. Group C was a control group. | This program focused on implementing a Person-centred Care Approach (PCC) in dementia care with an aim to improve staff’s attitudes towards dementia, job satisfaction and reduce burnout. The intervention period was 18 months. The program consisted of six phases:   1. Training of two caregivers per unit (three-day course) in PCC and observation and feedback skills. Preparation of the whole team. 2. Observations of care practices in the unit; by the two trained caregivers; 5-8 hours. 3. Data analysis and report writing by the trained caregivers. 4. Feedback of results to other staff. 5. Developing an action plan based on the gathered data and team’s feedback. 6. Implementation of the action plan.   Not reported who trained the staff. | n=290 care staff (nurses, social workers and nurse aids) in nine nursing homes in Germany; group A – n=81; group B – n=106; group C – n=103; exact number of nurse aids not reported. | **Job satisfaction**  A significant decrease in scores over time (less satisfaction) in Group A.  Scores increased in Group B and C; unclear if statistically significant.  **Burden**  A significant increase in scores over time (more burden) in Group A. Scores also increased in Group B and C; unclear if statistically significant.  **Staff attitudes**  A significant decrease in scores over time (worse attitudes) in all groups. | **Not applicable** |
| **Finnema, E. et al.** **(2005); Risk of bias: Moderate;**  **Comment:** Outcome assessors were not “blind”. Randomisation and concealment methods not reported. Perceived work-related stress was measured with the Organization and Stress Scale (Bergers et al., 1986); Stress was measured with the General Health Questionnaire (Goldberg and Hillier, 1979); work satisfaction was measured with the Dutch Work Satisfaction Scale (Boumans, 1990); absenteeism was measured as the number of days of absenteeism as reported by administration staff of the participating nursing homes. | | | | |
| A randomised controlled trial. Measurements were taken at baseline, and three and seven months later (intervention still active). | The integrated emotion-oriented care was a nine-month program delivered by nursing advisors.  All NAs received a two-day basic training. This course focused on NAs’ own experience, phases of ego-experience in demented residents, and application of empathic skills.  An advanced course was offered to five NAs on each ward. This course consisted of seven days spread over a period of seven months, and focused on acknowledging the residents’ experiences, making a life history, and being alert to how past may affect the present.  One staff per ward was trained to become an ‘adviser emotion-oriented care’. Motivated and enthusiastic staff with good interpersonal skills were selected to attend this last course. This course consisted of ten days, spread over nine months. This group was responsible for implementing the emotion-integrated care on their ward. No specific guidelines on how to implement the emotion-integrated care principles were given to advisers. | n=99 NAs in n=14 nursing homes in the Netherlands; n=46 in the experimental group and n=53 in the control group. | **Perceived work-related stress**  No significant changes found.  **Stress**  Subgroup analysis: a significant improvement (less stress reactions) was found for NAs in the experimental group who applied more emotion-integrated care at seven months than at baseline compared to NAs in the control group who also felt they had improved regarding emotion-integrated care. No other significant changes found. | **Absenteeism**  No significant changes found. |
| **Fragala, G. (2012); Risk of bias: High;**  **Comment:** This was a pilot study with a control group. Unclear and very limited information on most study design characteristics, study sample or study results. Outcomes were measured using a not validated survey. Staff attitude survey concerned staff’s morale and their perceptions on management’s commitment to providing a safe work environment. | | | | |
| A before and after study design with a control group; outcome measures taken at baseline and three months later (Intervention still active). | The program is a continuous initiative and focuses on addressing safe patient handling, preventing accidents, controlling losses, improving the quality of work life for staff, and quality of care for residents. The program can be delivered by a patient handling trainer. Staff who undergo the training, become expert users and serve as a resource to other staff. The program has a five-step framework:   1. Risk identification. 2. Identification of areas requiring change. 3. Formulating recommendations to eliminate hazards. 4. Implementation (including education, buy-in, and training). 5. Measuring effectiveness and encouraging ongoing improvement. | Nursing staff in a long term care facility in Massachusetts, US; n=24 nursing staff (including registered nurses, licences practical nurses, nurse aides, and other staff). | **Staff attitude**  Attitude scores were higher after at three months post-implementation than at baseline in the experimental group. No statistical test results were reported. | Not applicable. |
| **Fukuda, K., et al. (2018); Risk of bias: Moderate;**  **Comments**: Quasi-randomised. Groups had an important difference – residents in the intervention group were more independent. However, all other aspects look good, so the risk of bias is acceptable. Numbers of care staff groups not reported. Stress/burnout was measured with the Maslach Burnout Inventory (Maslach & Jackson, 1981). | | | | |
| A cluster quasi-randomised controlled trial. Data were collected at baseline and one-month after baseline. | The study used an intervention which was an educational program administered at baseline using printed educational material for the care staff called The Guidelines. The program was divided into two sections:  Section 1 was composed of a 30-min educational lecture providing an overview and covering the basic principles of BPSD. Section 2 consisted of a thorough, 90-min explanation of the proper way to use The Guidelines when BPSD occurs at a care facility. These two sections were intended to thoroughly introduce the method of using The Guidelines to the care staff. | n=400 care staff members (including care workers, nurses, OTs and psychologists) from 22 care facilities in Japan; n=214 staff in the intervention group and n=186 in the control group. Exact care workers numbers not reported. | **Stress/burnout**  No significant changes were found. | **Not applicable** |
| **Harman, B. (1998); Risk of bias: High;**  **Comment:** Very small sample size. No randomisation. Job turnover rates were collected from the administrative staff at the facilities. | | | | |
| A multisite, repeated measures, quasi-experimental group study. Outcome data was collected at baseline, and three and eight weeks later (one and six weeks post-intervention). | The paraprofessional preceptor program focused on delivering a one-to-one interactive training in which a preceptor and preceptee are involved in communication and goal setting during preceptee’s orientation in their new facility.  The preceptor had to undergo a six-hour educational program focusing on issues related to caring for a resident with dementia and providing a learning environment for preceptees. The program was delivered by the study investigator and was based on their experience working in dementia care. Main topics included: myths and realities of dementia, communication techniques, behaviour management techniques.  After completion of the program, the preceptors were paired up with preceptees for a two-week period of orientation. | n=11 CNAs in n=6 dementia special care units in Missouri and Indiana, US; n= 6 in the experimental group and n=5 in the control group. | Not applicable | **Job turnover**  No significant changes found. |
| **Hsieh, H. et al.** **(2009); Risk of bias: High**;  **Comment**: No randomisation. There were significant differences between study groups at baseline. 12 participants were not included in the analysis as they completed less than 6 out of 8 education sessions. Caregivers stress was measured with the Work Stressors Inventory Chinese Version (WSI) (Lin, 2000). Even though the study reports significant improvements in caregivers’ knowledge of gerontology and decline in caregivers’ elder abusive behaviours, no significant changes in caregivers stress were reported. | | | | |
| A case control before and after study design. Measurements were taken at baseline and 10 weeks later (on week post-intervention). | The intervention consisted of eight weekly 90-minutes sessions led by a trained graduate nurse. Each session was attended by 10-12 caregivers.  The sessions focused on education and mutual support. The program covered issues related to ageing, managing residents’ health problems, elder abuse, relaxation, stress management, and obtaining personal resources. The first 30 minutes of the session was delivered as a lecture, the next 40 minutes focused on sharing experiences and mutual support, and the last 20 minutes were dedicated to a group discussion. | n=112 caregivers (nurses and NAs) in n=4 nursing homes in Taiwan; n=2 experimental facilities and n=2 control facilities. | **Caregiver stress**  No significant changes were reported. | Not applicable |
| **Inker, J. et al. (2021); Risk of bias: Very high;**  **Comment:** No control group, before and after evaluation of a 52-week microlearning intervention for direct care staff, including certified nursing assistants, registered nurses and other staff. Not clear how many support workers were included. Job satisfaction was measured with the Nursing Home Nurse Aide Job Satisfaction Questionnaire (Castle, 2007); staff attitude – attitude towards dementia - was measured using the Dementia Attitude Scale (O’Connor & McFadden, 2010). | | | | |
| A before and after study with no control group. Measures were taken at baseline and post completion of the intervention. | The 52-week Microlearning intervention was delivered between April 2016 and March 2017. The weekly Microlearning lessons were made available online learning and accessible 24/7 via computer, tablet, and smartphone. The content included topics on Person-centred care, dementia-related challenges, active listening skills and other. It was created by the third author who used videos and exercises from the Centers for Medicare & Medicaid Services (CMS) Hand in Hand Toolkit. The 52 lessons  averaged 6 min and comprised brief content  with summary points and a two question quiz to assess  learning; they were organized into five topic areas and built upon each other although they could also stand alone.  Nursing homes also received weekly flyers highlighting the lesson. Users could go back to lessons and re-watch them as needed. Nursing homes were encouraged to identify “super users” who acted as champions for the Microlearning project, encouraging peers to complete the training and assisting with troubleshooting technical problems like computer access. Some nursing homes mandated participation, while others did not but used incentives to encourage participation.  Those who completed all the assigned lessons and passed the quizzes were awarded certificates of completion. | n=250 care staff members (including care workers, nurses, OTs and psychologists) from nine care facilities in Virgina, US. Exact numbers of nursing assistants not reported. | **Job satisfaction**  A significant improvement in job satisfaction was reported.  **Staff attitude (dementia attitude)**  A significant improvement in staff’s attitudes towards dementia was reported. | Not applicable |
| **Isaia, G. et al. (2011); Risk of bias: Very high;**  **Comment:** No control group. This was a hypothesis-generating study. Stress was measured with the Staff Stress Measure Dementia Care Scale (Gruetzner, 2001); only individual item improvements were reported; no total or subtotal scores comparisons. | | | | |
| A single group before and after study. Data was collected at baseline, and at 4 months post-intervention. | The program aimed to increase NAs knowledge about aged care, and to improve their work-stress coping skills. It lasted eight months. The program consisted of two hours of training per week; total of 32 hours of theoretical lessons and 32 hours of role playing and discussions.  The main topics of the lessons were dementia, behavioural management, nutritional issues in dementia, pain in dementia, communication skills, impact of dementia on residents’ lives.  The training was led by a senior physician, by a nurse experienced in dementia care, by a psychologist experienced in group training, by a nutritionist, and by a pain specialist. The senior physician and the nurse were available to participants for an additional one hour a week to discuss any issues and provide general support. | n=50 professional caregivers (NAs and registered nurses) in n=2 nursing homes in Italy. Exact number of NAs in the sample not reported. | **Stress**  Statistically significant improvements on nine out of 20 items were reported.  A significant decline on one out of 20 items was reported.  No other significant changes were found. | Not applicable |
| **Jung, D. et al. (2020); Risk of bias: Very high;**  **Comment:** Control group; however, control group’s characteristics were statistically significantly different from the experimental group, e.g., baseline job satisfaction scores were 34 in the control vs 39 in experimental. Job satisfaction was measured using the Job Satisfaction Scale for Clinical Nurses (Han & Mun, 1996). Self-esteem was measured using the Korean Nursing Assistant Self-Efficacy for Restorative Care Scale (Resnick & Simpson, 2003). | | | | |
| A before and after study with a control group. Measures were taken at baseline, and 6 and 12 weeks later. | The six-week Korean Function-Focused Care Program focused on educating nursing assistants about function-focused care, which aims to maintain the residents residual function and maximise their performance of activities of daily living. NAs in the experimental group initially attended a two-hour lecture on Function-Focused Care, and were then given teaching materials. Additionally, researchers were encouraging (or ‘coaching’) the NAs to implement their new skills in practice. The educational content focused on eating, dressing, using the toilet, walking, using assistive devices, and exercise. | N=50 long-term care workers in two long-term care facilities in Seoul, South Korea. There were 26 workers in the control group and 24 in the experimental. | **Job satisfaction**  No statistically significant changes were reported. MANCOVA found that the effect of the intervention on job satisfaction was statistically significant.  **Self-esteem**  No statistically significant changes were reported. MANCOVA found that the effect of the intervention on self-esteem was statistically significant. | Not applicable. |
| **Kuske, B. et al. (2009); Risk of bias: Low;**  **Comment:** High quality study. No obvious risk of bias identified. The findings were reported for the total sample (not for NAs only). Burnout was measured with the German version of the Maslach Burnout Inventory (Bussing et al., 2003); level of health complaints was measured with the Health Complaints List (Zerssen, 1976). Authors conclude that education is only the first step in improving everyday practice and argue the importance of focus on implementation of new knowledge. They also suggest more recognition of staff’s potential is necessary to allow staff to implement the new knowledge. | | | | |
| A cluster-randomised controlled trial. Data was collected at baseline, and three and six months later (immediately post and three months post-intervention). | The intervention aimed to improve nursing staff’s interactions with residents through improving staff’s knowledge on dementia, communication, and care skills.  The intervention was delivered over a period of 13 weeks (three months) in one-hour weekly sessions.  The intervention was delivered by a health and nursing scientist with applied experience in nursing.  One of the control groups, the relaxation group, received 13 weekly one-hour relaxation sessions. These relaxation sessions were delivered by a certified clinical psychologist. | n=96 nursing staff (nurses, NAs, occupational therapists, and other) in n=6 nursing homes in Germany; n= 38 staff in the experimental group (at least 15 NAs), n=30 staff in the relaxation group (at least 14 NAs) and n=28 staff in the control group (at least 7 NAs). | **Burnout**  No significant changes were found.  **Health complaints**  A significant decrease in health complaints for relaxation group.  No other significant differences were found. | Not applicable |
| **Li-Yu, W. et al.** **(2005); Risk of bias: High;**  **Comment:** Non-randomised controlled trial. The control group was made up of people who were not allowed (by their managers) to participate in the training. Small sample size. The groups were not similar at baseline. Work stressors was measured with the Work Stressor Inventory (Lin et al., 2002); | | | | |
| A before and after study design with a control group. Outcome data was collected at baseline and three months later (at completion of the intervention). | The purpose of this weekly empowering in-service training was to promote a sense of control and increase awareness of autonomy in foreign NAs. The series consisted of 10 one-hour meetings and was delivered by the study researchers.  The series started with a needs assessment process – this was an attempt to establish a trusting relationship between the NAs and researchers. The content of the following meetings included: physical assessment of resident, common diseases, emergency techniques, wound care, rehabilitative exercise, nutrition, stress management, communicable diseases protection for facilities, occupational risk protection, and physical exercise. | n=35 foreign NAs in n=10 long term care facilities in Taiwan; n=16 in the experimental group and n=19 in the control group. | **Work stressors**  A significant difference in “workload/scheduling” after the intervention – the work stress of “workload/scheduling” increased in the experimental group.  No other significant changes found. | Not applicable. |
| **MacDonald, C. (2007); Risk of bias: Very high;**  **Comment:** No control group. Data on turnover came for the facilities administrator and included historical comparison data. Authors report a 36% decrease in turnover in one of the participating facilities, and an average 17% decrease in the study sample. | | | | |
| A single group before and after study design. Outcome data was collected at baseline, and at 3, 6, 9 and 12 months later. | The intervention was an online e-learning solution for long term care workers to improve the quality of care and quality of life of residents.  The program consisted of eight modules related to healthcare issues in long term care facilities. They were delivered online and available 24/7. The topics included: abuse and neglect, elopement attempts, corporate compliance, infection control, nutrition and hydration, pressure ulcers, provision of basic care, restraints. | n=753 healthcare workers in long term care facilities in Illinois, US; n=312 CNAs (41%) | Not applicable | **Turnover rates**  A downward trend was indicated but no statistical tests were performed. |
| **Mackenzie, C. et al. (2003); Risk of bias: High;**  **Comment:** No randomisation. No clearly stated research question/hypothesis. Groups were not similar before the intervention (shifts worked: greater proportion on night shifts in the experimental group; additionally, self-efficacy appeared significantly different at baseline). Self-efficacy was measured with a measure developed for this study, not previously validated; burnout was measured with the Maslach Burnout Inventory (Maslach et al., 1996); satisfaction with teamwork and relationships with other caregivers was measured using the Interaction subscale from the Organisational Job Satisfaction Scale (Sauter et al., 1997). | | | | |
| A before and after study design with a control group. Data was collected at baseline, and immediately post- an at three months post intervention. | This program aimed to decrease nursing staff’s stress and burnout through enhancing their self-efficacy in managing challenging work situations.  The programs consisted of four modules: teamwork module, challenging resident behaviour module, family module, and a review module. Each module was two hours long, delivered once a week for a month. The modules started from a one hour didactic session, followed with one hour of experiential role-playing.  The knowledge and skills were reinforced with a training manual and placement of posters highlighting key strategies.  Total cost of the course was $4,396 USD. It was not reported who delivered the intervention. | n=41 nursing staff from n=2 units in a long term care facility in Toronto, Canada; n=28 staff in the experimental group (n=20 NAs) and n= 13 staff in the control group (n=10 NAs). | **Self-efficacy**  A significant difference at three-month follow-up favouring the experimental group was found.  **Burnout**  A significant treatment effect for personal accomplishment was found at post-intervention assessment, but not at three- month follow-up.  No other significant changes were found (emotional exhaustion and depersonalisation subscales).  **Satisfaction with teamwork/relationships with co-workers**  No significant differences were found. | Not applicable. |
| **Noel, M. et al. (2000); Risk of bias: High;**  **Comment:** No randomisation. Difference between control and treatment groups. Study used data from before the study period. Turnover and absenteeism data was provided by the facilities. Turnover was expressed as the total number of terminations divided by the total number of budgeted FTEs (full time equivalents) in the last year. Absenteeism was expressed as the total number of absences divided by the total number of budgeted FTEs. | | | | |
| A before and after study design with a control group. Data was collected at 12 months and compared with historic data. | An in-house education program based on the North Carolina State curriculum for CNAs was developed and evaluated. It focused on improving the orientation for new staff, adding a mentorship component and formal instruction by a nurse educator. The nurse educator was employed to work 20 hours a week in each facility for the full 12 months.  The program included structured incentives for good performance and encouragement of licensed staff to include CNAs in the facilities’ interdisciplinary teams.  The program also attempted to stabilise staff to resident ratios. | n=3 long term care facilities in North Carolina, US. | Not applicable | **Turnover**  A significant drop in turnover rates in one experimental facility, and a non-significant drop in the other experimental facility. The rates in the control group remained stable.  **Absenteeism**  No significant differences were found. |
| **Resnick, B. et al. (2004); Risk of bias: Very high;**  **Comment:** No control group. This was a pilot study; the final intervention was evaluated in the Resnick et al. (2009) study. Job satisfaction was measured with the Job Attitude Scale (Helmer et al., 1995). | | | | |
| A single group before and after study. Data was collected at baseline, and at four and 12 months later (intervention was still active). | The Res-care intervention was a self-efficacy-based motivational intervention. It focused on teaching the nursing assistants (NA) the Res-Care philosophy and skills (tier 1) and motivated the NA and helped them motivate and engage the residents in functional and physical activities. Tier 1 included six weekly 30-minute sessions led by two advanced practice nurse. Tier 2 focused on ongoing motivational support from a ‘champion’ Res-care nurse coordinator, over a 12-month study period. The Res-care ‘champion’ was providing ongoing supervision and motivational support, helped the NA prepare plans for the residents, and served as an interface between the NA and all other involved stakeholders. They also provided education session with respect to integrating Res-care into practice. | n=13 NAs from a nursing home in Maryland, US. | **Job satisfaction**  No significant changes were found. | Not applicable |
| **Resnick, B. et al.** **(2009); Risk of bias: Moderate;**  **Comment:** Randomisation method, concealment method and “blinding” not reported. Evaluated a range of outcomes unrelated to this review (e.g. intervention’s feasibility and uptake). Job satisfaction was measured with the Job Attitude Scale (Helmer et al., 1995). | | | | |
| A cluster-randomised controlled trial. Measurements were taken at baseline, and four and 12 months later (intervention still active). | The Res-care intervention was a self-efficacy-based motivational intervention. It focused on teaching the nursing assistants (NA) the Res-Care philosophy and skills (tier 1) and motivating the NA and helping them motivate and engage the residents in functional and physical activities. Tier 1 included six weekly 30-minute sessions led by advanced practice nurses. Tier 2 focused on ongoing motivational support from a ‘champion’ Res-care nurse coordinator, for 20hr a week over a 12-month study period. The Res-care ‘champion’ was providing ongoing supervision and motivational support, helped the NA prepare plans for the residents, and served as an interface between the NA and all other involved stakeholders. They also provided education session with respect to integrating Res-care into practice. | NAs in nursing homes in Maryland, US; n=283 in n=6 experimental sites, n=273 in control sites | **Job satisfaction**  Experimental group improved in job satisfaction from baseline to 12 months, whereas control group remained stable. No other significant changes reported. | Not applicable |
| **Tannazzo, T. et al.** **(2008); Risk of bias: Very high;**  **Comment:** A single group design. General job satisfaction was measured with the General Job Satisfaction scale (Hackman & Oldham, 1974); intrinsic and extrinsic job satisfaction was measured with the Grau Satisfaction Scale (Grau et al., 1991). | | | | |
| A before and after study design. Measures were taken at baseline, and at three (immediately post-intervention) and seven weeks later. | The treatment focused on training CNAs to manage difficult behaviours in patients with dementia. The training includes three 7.5-hour days over three weeks. The training curriculum includes six modules:   1. Putting the person first (recognising patient needs). 2. The environment (risk factors, wandering and decreasing wandering occurrence). 3. Enhancing the bathing experience. 4. Assisting with activities of daily living. 5. Mealtimes and the person with dementia. 6. Meeting the challenges of catastrophic reactions (dealing with patient’s anxiety, paranoia, hallucinations).   It was not reported who delivered the training. | n=301 CNAs in n=3 nursing homes in New York City area, US; | **General job satisfaction**  No significant changes.  **Intrinsic job satisfaction**  A significant increase in intrinsic satisfaction between immediately post-intervention and four weeks later. No other significant changes reported. | Not applicable |
| **Tynan, C. et al. (1984); Risk of bias: Very high;**  **Comment:** No control group. Turnover was reported as the total number of staff who left the facility between January and September. | | | | |
| A single-group before and after study design. Outcome measurement were collected at baseline and one year later. | The program aimed to prepare new staff better through an orientation program. The program focused on providing educational and psychological preparation and was based on principles of human relations and behavioural theories.  The first of the three parts of the program was a half a day presentation about the facility’s philosophy, structure, policies, facility layout, etc. This part was delivered by the administrative director and assistant director of the facility.  Part two was called ‘Sensoriperceptual Experience’. This part a 1.75-hour workshop delivered by a staff development instructor. During the workshop, the employees had to complete selected tasks of daily living while wearing a range of props to simulate having a disability. This was followed by a group discussion on the abovementioned tasks, on ageing, and on communication with elders.  Part three was a two-day workshop on basic nursing skills, delivered by a staff development instructor. This workshop covered a range of skills essential in the NA’s job.  Some educational resources were made available to all staff, including guidelines, lists of medications and their actions and side effects, and other.  Following completion of the three parts, the NAs were assigned to a clinical unit and buddied up for two days with a ‘reliable employee’ whose job was similar to theirs. | One long term aged care facility in Arizona, US. Total number of NAs not reported. | Not applicable | **Turnover**  No statistical tests were performed.  Downward trend was reported with the total number of terminations dropping from 47 to 28. Total number of NAs on the payroll was not reported. |
| **Wells, D. et al.** **(2000); Risk of bias: High;**  **Comment:** Intervention and control groups were significantly different at baseline (educational attainment, and employment status). Concealment method not reported. Not all trainers and outcome assessors were “blind”. Only n=1 active cluster vs n=3 control clusters. Caregiver’s level of stress was measured with the Hassles Subscale of the Nurses Hassles and Uplifts Scale (Craig, 1995). | | | | |
| A cluster-randomised trial with data collected at baseline, and at three and six months post-intervention. | Caregivers in the experimental unit were educated to provide the abilities-focused program of care. The program consisted of five 20-30-minutes sessions. The program focused on: a) effects of dementia on the social abilities and on the self-care abilities. Strategies addressing issues within those abilities were taught to the caregivers. For example, how to use reflexes to facilitate bathing or dressing. Each session was repeated until all caregivers in the unit received all five sessions. Reinforcement sessions were provided every second week for three months, and then monthly until completion of the study. In these sessions caregivers were sharing their experiences in implementing the new skills.  The training was delivered by the study researchers. | n=44 caregivers in n=4 nursing homes in Canada; n=16 caregivers in the experimental unit and n=28 caregivers in the control units. | **Caregiver’s level of stress**  No significant changes were found. | Not applicable. |
| **Zimmerman, S. et al. (2010); Risk of bias: Moderate;**  **Comment:** Randomisation, concealment and ‘blinding’ methods not reported. Work stress was measured with the Work Stress Inventory (Shaefer & Moos, 1993); job satisfaction was measured with the Staff Experience of Working with Demented Residents Questionnaire (Astrom et al., 1991); role recognition was measured with the Benjamin Rose Relationship with Supervisor Scale (Kiefer et al., 2009). | | | | |
| A nested cohort group-randomised trial. Data was collected at baseline, and immediately post- and three months post-intervention. | This was a dementia care training program. The training for NAs differed slightly from the supervisors training. The NAs training consisted of three modules delivered in four sessions over a period of six weeks.  First module, Learning to lead – Building a vision, was a one session module.  About dementia – Improving communication, was the second one-session module, and focused on communication challenges and strategies to improve communication.  The third module, Reducing pain, was a two-session module. It focused on sources and expressions of pain, and strategies to report, prevent and respond to pain.  It was not reported who delivered the intervention. | n=662 nursing staff (NAs, nurses, medication assistants, LPNs) from 16 long term care facilities in US; n=291 staff in the training group (n=165 NAs) and n=371 in the control group (n=208 NAs) | **Work stress**  A significant increase in work stress score for NAs at three-month follow-up in the training group. No other significant changes found.  **Job satisfaction**  Secondary analyses: A significant difference was found between facilities with low intervention fidelity compared with facilities with high intervention fidelity, favouring the high intervention fidelity.  No other significant changes were found.  **Role recognition**  No significant changes were found. | Not applicable |

## Table 2. Interpersonal skills-based interventions summary table.

| **Design** | **Intervention** | **Population** | **Psychosocial outcomes** | **Turnover-related outcomes** |
| --- | --- | --- | --- | --- |
| **Boersma, P., et al. (2017); Risk of bias: High;**  **Comments:** No randomisation. Groups were not similar. Less than a half of participants were included in the final analysis. Job satisfaction was measured with the Leiden Quality of Work Questionnaire (Van der Doef and Maes, 1999). | | | | |
| A before and after study with a control group. Data was collected at baseline and nine months later. | This program used the Veder Contact Method (VCM), which combines methods such as reminiscence, validation, emotion-oriented care, and neuro-linguistic programming. VCM seeks to improve contact between patient and caregiver. It follows a fixed procedure of: (1) greeting the resident, (2) appealing to long-term memory, (3) communication about the present time, and (4) saying goodbye.  The training includes:   1. Five 3-hour group sessions on knowledge transfer and skills training. 2. Three 3-hour on-the-job coaching sessions focusing on behavioural observation and direct feedback.   The training was delivered by VCM experts over a period of nine months. | n=111 nursing staff from n=4 nursing homes in the Netherlands. n=75 staff in the experimental group (n=27 NAs and nursing hostesses); n= 36 staff in the control group (n=5 NAs and nursing hostesses). | **Job satisfaction**  No significant changes were found. | **Not applicable** |
| **Broughton, M. et al.** **(2011); Risk of bias: High;**  **Comment:** Participants in the intervention group had significantly more years of experience in their current role. Treatment allocation was not random. Not all subjects analysed in the groups they were initially allocated to. Satisfaction was measured with the Positive Aspects of Caregiving (Tarlow et al., 2004). Authors reported that the qualified nurses’ and NAs satisfaction scores were significantly higher than activities staff’s scores. However, only the nurses scores improved significantly as a result of the intervention. | | | | |
| A before and after study design with a control group. Data was collected at baseline and at 3 months post-intervention. | The experimental group received training in supporting memory and communication in people with dementia. The training provides the NAs with two evidence-based strategies.  The training is delivered in one 90-minute session. The main component of it is a 50-minute DVD exemplifying the two strategies. The DVD screening is followed by an expert commentary from a psychologist and speech pathologist. Participants were given a booklet summarising the DVD material and a lanyard card listing the strategies. Also, posters listing the strategies were placed around the facility. | n=52 nursing staff (NAs, nurses, activities staff) in n=4 nursing homes in Queensland, Australia; n=37 in the experimental group (n=22 NAs) and n=15 in the control group (n=11 NAs). | **Satisfaction**  No significant differences were found for NAs. | Not applicable. |
| **Covert, B. (2007); Risk of bias: Very high;**  **Comment:** No randomisation. No control group. Turnover rates were based on the data collected from the participating facilities administrators and expressed as the number of terminations within preceding 28 days divided by the number of NAs on payroll in the same period. Sick day utilisation was also based on the data collected from the administrators and expressed as the sum of sick days taken in the preceding 28 days divided by total days worked in the same period. | | | | |
| A before and after study design. Outcome measurements were collected at baseline, and at 4 weeks post-intervention. | The Customer Service Training program aimed to lower the turnover and sick leave utilisation through teaching communication and interpersonal skills. It was delivered by an expert in designing and instructing customer service training.  The training was delivered in a single day and took approximately twelve hours. It consisted of three main modules: 1) attitude training (improving team work and empathising with the residents), 2) action training (communication skills), and 3) accountability training (to encourage and increase good behaviour in NAs). | n=97 NAs in n=2 nursing homes in Ohio, US. | Not applicable | **Turnover rates**  No significant changes found.  **Sick leave utilisation**  No significant changes found. |
| **Franzmann, J. et al. (2016); Risk of bias: High;**  **Comment:** No randomisation. A lot of the study design characteristics not reported. Study groups were not similar. The experimental group was made up mostly of registered nurses (62%). Mental stressors at work were measured with the SALSA (Rimann & Udriss, 1997); the occupational mental stress was measured with the Short Version BHD (Hacker & Reinhold, 1999). | | | | |
| A before and after study design with a control group. Data was collected at baseline, and at four weeks and six months post intervention. | The program uses a train-the-trainer approach and views the trainers as moderators rather than teachers. The main aim of the program is to improve NAs communication skills with people with dementia.  The trainers were called multiplicators and were trained over a period of six months, for 120 minutes every two weeks. The training qualified the multiplicators to design, deliver and evaluate training sessions for their colleagues. During these sessions, the multiplicators were passing on their knowledge on communication skills.  It was not reported who trained the multiplicators. However, a training manual is available.  It was not reported how many sessions a month were the multiplicators organising for their colleagues. | n=116 geriatric caregivers (CNAs, NAs, registered nurses) in n=14 nursing homes in Germany; n=31 caregivers in the experimental group (n=13 NAs and CNAs) and n=81 caregivers in the control group (n=47 CNAs and NAs). | **Mental stressors at work**  A significant decrease in the experimental group when compared to control group at six-month follow-up.  No other significant changes reported.  **Occupational mental stress**  A significant decrease in the experimental group when compared to control group at six-month follow-up.  No other significant changes reported. | Not applicable |
| **Haberstroh, J. et al. (2011); Risk of bias: High;**  **Comment:** No randomisation. Not clear how many nurses vs NAs in the sample. Four comparison groups (control, only first training session, only second training sessions, both training sessions); not reported how many people in each group. Job stress was measured with the Occupational Stress Screening for Human Service Providers (Hacker & Reinhold, 1999). | | | | |
| A before and after study design with a control group. Data was collected at baseline, and seven weeks later (post-intervention). | This was a communication training program for professional caregivers in dementia care.  The program consisted of two training sessions, eight-hour long each. They were held with an interval of two weeks. First session focused on communication with residents with dementia. Second session focused on communication with colleagues. Not reported who delivered the sessions. | n=53 geriatric caregivers from n=6 nursing homes in Germany. n=4 comparison groups (group sizes not reported). | **Job stress**  A significant treatment effect favouring the experimental group was reported. | Not applicable. |
| **McCallion, P. et al.** **(1999); Risk of bias: Moderate;**  **Comment:** Randomisation and concealment methods not reported. Only two clusters. Turnover was measured as the number of people leaving the facility in the last calendar quarter. The authors discussed the importance of booster sessions to ensure continued implementation of the program (communication skills). The participating facilities were able to continue the program using existing staff and resources. | | | | |
| A study using a cluster (care units) randomised controlled trial design. The data was collected at baseline (immediately post-intervention), and at three, six and nine months post-intervention. | The Nursing Assistant Communication Skills Program consisted of five 45-minute group sessions and four 30-minute individual sessions. The individual sessions served as a way to personalise the training, practice and provide feedback about skills taught in the group sessions. Also, they served as make-up sessions for NAs who were unable to attend the group sessions.  The program was delivered by a master’s level social worker with experience in working with residents with dementia. The social worker was required to do some background reading and then attend a training. Their training focused on: a) stages of dementia, b) verbal and nonverbal communication strategies in dementia, c) developing and using memory aids, d) approaches to training NAs, agendas for group and individual sessions, and how to provide feedback.  During the group and individual sessions, the social worker followed the agenda and taught the NAs about the abovementioned topics. | NAs in n=2 nursing homes in New York area, US; n=39 NAs in experimental group and n=49 NAs in control group. | Not applicable | **Turnover**  A significant decline in turnover rates from baseline to six months in the experimental group when compared to the control group. No other significant changes. |
| **Passalacqua, S. et al. (2012); Risk of bias: Very high;**  **Comment:** No control group. Outcome measures were delivered in English. However, some of the participants found the measures difficult to understand at pre-intervention test (42% were not native English speakers). The authors decided to truncate the measures and used them at post-intervention assessments, without previously validating them. Happiness was measured with two items from the Shortened Depression-Happiness Scale (Joseph et al., 2004); burnout was measured with the Emotional Exhaustion Subscale of the Maslach Burnout Inventory (Maslach & Jackson, 1981). | | | | |
| A single group before and after study. Data was collected at baseline; and at approximately 14 weeks later (10 weeks post-intervention). | The program aimed to teach NAs appropriate communication skills to foster person-centred care. It was delivered by two researchers with expertise in gerontology and patient-provider communication. The program was offered in four one-hour sessions over a period of four weeks.  Each workshop focused on specific topics related to communication. Week 1 focused on “valuing people; week 2 on “individualised care”; week 3 on “personal perspectives”; and week 4 on “Social environment”. | n=26 paraprofessional caregivers in a long term care facility for people with dementia in Southwest, US. | **Happiness**  No significant changes were found.  **Burnout**  No significant changes were found. | Not applicable |
| **Pillemer. K. et al.** **(2003); Risk of bias: Moderate;**  **Comment:** Randomisation method, concealment method and “blinding” not reported. Evaluated some outcomes related to the interventions feasibility and uptake. Relatively high drop out in the experimental group (40 vs 27%). Depressive symptomatology was measured with CES-D (Radloff, 1977); burnout was measured with the Maslach Burnout Inventory for nursing staff (Pillemer & Moore, 1989); intention to quit was measured by a single item asking about likelihood of quitting job in the next 12 months (not validated). | | | | |
| A randomised controlled trial with measurements at baseline, and at two and six months post-intervention. | The main aim of the program was to increase cooperation and effective communication between staff and family members of residents. The program consisted of two parallel workshop series: one for CNAs, and one of family members of residents; and a combined two-hour session for staff and families at the end of the workshop.  The CNA workshop consisted of 9 sessions and took approximately seven hours on one day. The sessions were built around three main concepts:   1. Active listening. 2. Giving feedback. 3. “I-messages”.   The program was delivered by trainers with extensive leadership experience. | Nursing staff in n=20 nursing homes in Central New York region, US; n= 256 in experimental group (68% CNAs) and n=399 in control group (66% CNAs). | **Depressive symptomatology**  No significant changes reported.  **Burnout**  No significant changes observed. | **Intention to quit**  A significant difference between experimental group (decline in intention to quit) and control group (increase in intention to quit). |
| **Robison, S. et al. (2007); Risk of bias: Moderate;**  **Comment:** This program was a specialised dementia unit adaptation of the Pillemer et al. (2003) study. Randomisation, concealment and blinding methods not reported. Participants who did not complete follow-up assessments were excluded from the outcomes analysis (not intention-to-treat approach). Depression was measured with the Center for Epidemiologic Studies – Depression (Ross & Mirowsky, 1989); burnout was measured with the Depersonalisation Subscale of the modified Maslash Burnout Inventory (Pillemer & Moore, 1989); job satisfaction was measured with the Generic Job Satisfaction Scale (MacDonald & MacIntyre, 1997); intention to quit was measured with a not previously validated single item; job stress was measured with a not previously validated single item. | | | | |
| A randomised controlled trial. Data was collected at baseline, and at two and six months post-intervention. | This program was a specialised dementia unit adaptation of the Partners in Caregiving Program (Pillemer et al., 2003). Modifications included a new module on understanding dementia and behavioural symptoms of dementia, as well as a number of case studies illustrating specifics of working with people with dementia.  The program consisted of two parallel workshop series: one for nursing staff, and one of family members of residents; and a combined two-hour session for staff and families at the end of the workshop. The workshop consisted of 9 sessions and took approximately seven hours on one day. The sessions were built around three main concepts:  1. Active listening.  2. Giving feedback.  3. “I-messages”.  The program was delivered by trainers with extensive leadership experience. | n=384 nursing staff (CNAs, LPNs, registered nurses) in n=20 nursing homes in Connecticut, US; n=184 in the experimental group staff (n=134 CNAs) and n=200 staff in the control group (n=146 CNAs). | **Depression**  No significant changes were found.  **Burnout**  No significant changes were found.  **Job satisfaction**  No significant changes were reported.  **Job stress**  No significant changes were reported. | **Intention to quit**  No significant changes were reported. |
| **Schrijnemaekers, V. et al.** **(2003); Risk of bias: High;**  **Comment:** No clear research question/hypothesis. Randomisation and concealment methods not reported. The outcome assessors were not ‘blind’. There were differences between groups at baseline. Job satisfaction was measured with the Maastricht Work Satisfaction Scale for Healthcare (MWS) and the Short Maastricht Work Satisfaction Scale for Healthcare (short MWS) (Landeweerd et al., 1996) and with three other not validated items; burnout was measured with Maslach Burnout Inventory (Schaufeli et al., 1993); sick leave utilisation in last three/six months was reported by the participants as days off sick in that time period; work situation was measured with a single not validated item. | | | | |
| A randomised controlled trial study. Outcome data was collected at baseline, and at three, six and 12 months post-intervention. | The emotion-oriented care training aimed to reduce burnout and sick leave and improve job satisfaction in professional caregivers. It consisted of three parts. Firstly, all employees at the facility attended two clinical lessons which informed the participants about the general ideas of emotion-oriented care.  The second component of the program was delivered by ‘a highly experienced and motivated teacher’ over a period of six days. The first four days were given at intervals of two weeks, and the last two days were given at an interval of four weeks. The sessions were attended by eight caregivers from each facility. They had to be ‘key figures’ in the daily care for residents and be able to implement the emotion-oriented care approach in their facility. The participants were taught about dementia, communication skills, and understanding the residents’ perception of the environment.  The third component of the program were three supervisions meeting (half-a-day each). They were held over a period of four months, after completion of the second component. They served as a platform to discuss goals, agreements and evaluations of the program. | n=300 professional caregivers in n=16 homes for elderly in the Netherlands; n=155 caregivers in the experimental units and n=145 in the control units. | **Job satisfaction**  Linear trend analysis (average change per month): a significant difference in ‘opportunities for self-actualisation’ subscale, in the short MWS, and in ‘satisfaction with contact with residents’ in favour of the experimental group. These differences appeared mainly after 12 months of follow-up.  Some other significant changes (satisfaction with ‘head of the ward’, ‘quality of care’, ‘contact with colleagues’, and ‘opportunities for self-actualisation’) in favour of the experimental group at 12 months were reported, but no data was shown.  **Burnout**  Linear trend analysis (average change per month): a significant difference in ‘personal accomplishment’ in favour of the experimental group. This difference appeared after 12 months of follow-up. No other significant changes reported.  **Work situation**  No significant differences were found. | **Sick leave utilisation**  No significant differences were found. |
| **Sprangers, S. et al.** **(2015); Risk of bias: High;**  **Comment:** Very small sample size. No randomisation. Intervention was delivered by the researchers. Job satisfaction was measured with Utrecht Work Engagement Scale (Schaufeli & Bakker, 2004); caregiver distress was measured with the Dutch version of the Neuropsychiatric Inventory Questionnaire (Kaufer et al., 2000). | | | | |
| A before and after study with a control group. Outcome data were collected at baseline (pre-intervention) and eight weeks later (post-intervention). | The intervention focused on teaching the NAs communication skills suitable for different types of dementia. The number of sessions for each nursing aide (NA) depended on their communication skills checklist score – either one (better scores) or two (worse scores) sessions.  The training session were delivered by the researchers. NAs were observed during their interactions with residents. After these observations, NAs received feedback on their communication. They were encouraged to keep using the effective strategies that they had used, and to start using new skills they had not used. The use and purpose of new skills were explained. If they used negative strategies, it was explained to them why they should not use these strategies. | NAs in a nursing home in the Netherlands. n= 24 NAs. Number of people in control and intervention groups not reported. | **Job satisfaction**  No significant changes.  **Caregiver distress**  A significant improvement over time in the intervention group when compared to control group. | Not applicable. |
| **Teri, L. et al. (2005); Risk of bias: High;**  **Comment:** Randomisation, concealment and blinding methods not reported. Very small sample. Primary outcome, job satisfaction, not measured with a previously validated instrument. | | | | |
| A small randomised controlled trial with data collected at baseline, and eight weeks later (immediately post-intervention). | The program was based on an integrated model of person-environment fit and social learning theory. The program taught staff how to identify factors within their environment, and within their interactions with residents, that can be modified to improve the care they provide and reduce resident distress. The program focused on: a) basic information about dementia and its consequences, b) communication skills, c) ‘pleasant events’ for residents, and d) managing residents’ distress.  The program was delivered over a period of two months, as two half-day workshops and four individualised sessions. Not reported who delivered the intervention. | n=25 direct care staff in n=4 assisted living facilities in Washington, US; group sizes not reported. | **Job satisfaction**  No significant changes were found. | Not applicable |

## Table 3. Team-building interventions summary table.

| **Design** | **Intervention** | **Population** | **Psychosocial outcomes** | **Turnover-related outcomes** |
| --- | --- | --- | --- | --- |
| **Catanzaro, D. (1992); Risk of bias: High;**  **Comment:** A wide range of outcomes were measured (many referring specifically to satisfaction), yet statistical significance levels were not adjusted, with some analyses using *p*<0.1 as level of statistical significance. Some differences between groups at baseline. very high dropout, approx. 60% (due to high staff turnover); those people were not included in the final analysis. Small final sample (n=25 at second post-test). Perceived influence was measured with a not validated tool (showed good internal reliability in the current study); satisfaction with influence was measured with the Satisfaction with influence questionnaire (Rafaeli, 1985); organisational commitment was measured with the Organisational Commitment Questionnaire (Mowday et al., 1979); turnover intention was measured with the Michigan Organisational Assessment Questionnaire (Cammann et al., 1983); role conflict and role ambiguity were measured with the Role Conflict and Ambiguity Scale (Rizzo et al., 1970); satisfaction of higher-order needs was measured with the Need Satisfaction Scale (Lawler & Hall, 1970); satisfaction with service role and satisfaction with organisational policies were measured with the Minnesota Satisfaction Questionnaire (Weiss et al., 1967); turnover rates were obtained from the participating facilities’ administrators. | | | | |
| A before and after study design with a control group. Data was collected at baseline, and 34 weeks and 50 weeks later (26 and 42 weeks post-intervention). | The program consisted of weekly one-hour problem-solving meetings. They were attended by NAs and other members of the care team, over a period of seven weeks. The meetings were attended by four to six people.  Meeting 1 was used to identify a range of problems affecting the NAs daily work.  Meetings 2 and 3 were used to examine causes contributing to the problems identified in meeting 1, and to rank the problems in terms of their impact on residents or families satisfaction.  Meeting 4 was used to discuss the top two serious problems and brainstorm solutions.  Meeting 5 was used to evaluate possible solutions, and to select one or two best solutions to each problem.  During meeting 6, the NAs developed an action plan of how to implement the previously chosen solutions.  Meeting 7 was used to finalise the action plan.  The action plans were then presented to the management for their approval. The approved action plans were then implemented by NAs within the facility.  Meeting 8 was a ‘Behaviour modelling training sessions’, where a training film was used to depict handling common customer complaints. Meeting 8 was 90-minute long and was attended by two-three NAs. During this session, the NAs practiced the behaviours presented in the film, and received feedback from the facilities Director of Nursing. | n=72 NAs in n=4 nursing homes in Virginia, US; n=41 in the experimental group and n=31 in the control group. | **Perceived influence**  Some significant findings reported but unclear in which direction.  **Satisfaction with influence**  No significant differences were found.  **Role conflict and ambiguity**  No significant differences were found.  **Satisfaction with higher-order needs**  No significant differences were found.  **Satisfaction with service role**  No significant differences were found.  **Satisfaction with organisational policies**  No significant differences were found. | **Organisational commitment**  No significant differences were found.  **Turnover intention**  A significant improvement in the experimental group.  **Turnover**  No findings reported. |
| **Hegeman, C. et al. (2007); Risk of bias: Study 1 High, Study 2 Very high (before and after study);**  **Comment:** Study 1 - no randomisation. Groups were not similar at baseline. Sample sizes not clear. This paper reports the results from two studies evaluating the same intervention. Retention rates were defined as the percentage of CNAs who, hired at the beginning of the project, still remained at the facility. In Study 2, one of the groups was not included in the analysis due to “insufficient data collection”. | | | | |
| Study 1: A before and after study with a control group.  Data was collected at baseline, and at three months post-implementation (intervention still active).  Study 2: A before and after study with no control. Data was collected at baseline, and at three and six months post-implementation (intervention still active). | Same for both studies  This peer mentoring program aimed to improve CNA retention rates by improving orientation processes.  Mentors were trained in a six-hour workshop consisting of a range of mini-lectures. Topics included: mentor’s role, tools for mentoring, communication skills, importance of compassion, importance of attitude, leadership skills. Following the initial training, the mentors also received three three-hour booster sessions. These focused on reviewing the abovementioned content, and also on stress management, time management, adult education, and death and dying.  Each mentor is then paired-up with a new CNA for approximately four weeks. Mentors role is to model correct care skills, positive attitudes, and time management.  It was not reported who delivered the mentors training. | Both studies were conducted in New York, US.  Study 1:  CNAs in n=10 nursing homes in the experimental group and n=6 nursing homes in the control group.  Study 2:  CNAs in n=15 nursing homes; divided into three groups receiving the same intervention. Not clear how many facilities in each group. | Not applicable | **Retention rates**  Study 1:  A significant increase in the experimental group. No significant findings for the control group.  Study 2:  No report on pre-test vs post-test results. Significantly lower retention rates at six months compared to three months post-implementation.  No other significant findings reported. |
| **Howe, E. (2014); Risk of bias: Very high;**  **Comment:** No control group. Quality of work life was measured with the Quality of Work Life Survey (QWL) (Krueger et al., 2002). The study had a qualitative component evaluating participants’ feelings of empowerment and reported that participants experienced an increased sense of empowerment as a result of the intervention. | | | | |
| A single group before and after study design. Outcome measurements were collected at baseline and 12 weeks later (8 weeks post-intervention). | The Long Term Care Team Talk program consisted of a debriefing strategy, which involved an informal and brief but routine team meetings at the end of the shift to discuss the work day. The daily meetings were five-minute long and took place over a period of one month. The meetings were led by a rotating schedule of CNAs and focused on three aspects: 1) things that went well today, 2) things that can be improved, 3) what’s needed in order to improve.  Participants of the meetings included CNAs, nurses and other interdisciplinary team members as invited by the CNAs. | n=15 nursing staff in a long term care facility in New York, US; n=8 CNAs and n=7 nurses. | **Quality of work life**  A significant difference (improvement) in co-worker & supervisor support subscale was found between before and after scores.  The authors reported significant difference in the characteristics of the unit subscale scores. However, these were reported as significant at p<0.1 level.  No other significant differences were found. | **Intention to quit (QWL subscale)**  The authors reported significant difference in the intention to quit/transfer subscale scores. However, these were reported as significant at p<0.1 level. |
| **Petterson, I. et al. (2006); Risk of bias: Very high;**  **Comment:** No control group. Not clear how many residential care nursing assistants took part. A range of outcomes were measured but it is unclear what tools were used and the only information on the tools psychometric properties is regarding their internal reliability (based on the study sample). These outcomes included: workload (e.g. work demands, physical workload), staff resources (e.g. support from management, job satisfaction, control), health and well-being (e.g. stress symptoms, well-being), health resources (e.g. coping, mastery). | | | | |
| A single group before and after study design. Outcome measurements were collected at baseline and 18 months later (immediately post-intervention). | This 18-month intervention focused on empowering healthcare staff in elderly care and improving their work and health conditions.  In the first phase of the intervention, “Train the trainer” approach was used to facilitate participation. Selected NAs were trained one full day a week for ten weeks as leaders of the project in their own unit. The leaders were supported by each unit’s management and staff unions. The leaders training involved: sessions on quality of care and ethics, session on work quality, sessions on communication, coping, oral presentations and role-playing. As a motivational incentive, the leaders were given a pay rise.  In the second phase, the leaders returned to their units as trainers and managed competence circles where they were transferring the programme content to their colleagues. The circles were organised weekly for five weeks and took 6 hours each. These sessions were led by the leaders and supervised by geriatric teachers.  In the third phase, all units started their own projects addressing local issues identified in phase two. For example, orientation programs, quality improvement, nursing routines, etc. | n=200 nursing staff (80% were NAs, 20% were nurses and other staff) in n=14 care units in Sweden; n=127 nursing home staff and n=73 home carers. | **Workload**  No significant differences were found.  **Staff resources**  A significant decline in ‘learning and development’.  No other significant differences were found.  **Health and well-being**  Significantly worse scores on ‘psychosomatic symptoms’ and ‘musculoskeletal symptoms’.  Significant improvement in general health.  **Health resources**  No significant differences were found. | Not applicable. |
| **Pillemer, K. et al. (2008); Risk of bias: Moderate;**  **Comment:** Randomisation, concealment and ‘blinding’ methods not reported. Turnover rates data was provided by the facilities and expressed as a number of all CNAs who left the facility in the preceding 6 months divided by the average number of CNAs on the payroll during that period. Job satisfaction was measured using the Generic Job Satisfaction Scale (MacDonald & MacIntyre, 1997); stress was measured with one not previously validated item; job commitment was measured with one not previously validated item. The intervention led to a significant decrease in turnover rates of CNAs. The authors suggest reconceptualising the intervention from a retention specialist to a retention team. They also argue the importance of booster sessions. | | | | |
| A randomised controlled trial with data collected at baseline, and at six and 12 months later (intervention still active). | The program focused on training one CNA in each facility to become a Retention Specialist (RS) and to implement a range of retention strategies within their facility. This person received tools and ongoing support to carry out needs assessments, implement a range of retention strategies, evaluate the impact of their efforts and modify their approach as needed. This person served as the key internal advisor regarding retention programs.  The first component of the program, the RS training, consisted of three days. It focused on promoting retention practices (retention issues, creating climate for retention, diagnosis of retention, implementing retention programs) and on specific retention programs (e.g. mentoring programs, respect and recognition programs, programs to improve interpersonal skills).  The second component of the program was the ongoing technical assistance which was provided by the project staff. It involved access to retention-related resources online, and via telephone contact and print materials.  The third component of the program was to leverage community resources through establishing a ‘Living well, living healthy’ information kiosks. The kiosks were easily available to all facility staff and covered topics related to personal issues (e.g. financial well-being or health lifestyle).  It was not reported who delivered the program. | n=762 CNAs in 30 nursing homes in New York area and Connecticut, US; n=379 CNAs in the experimental group and n=383 CNAs in the control group. | **Job satisfaction**  No significant differences were found.  **Stress**  No significant differences were found. | **Turnover rates**  A significant decrease in turnover at 12-month assessment.  A non-significant decrease at 6-month assessment.  **Job commitment**  A significantly different change between six and 12-month assessments favouring the experimental group. |
| **Torsney, K. (2000); Risk of bias: High;**  **Comment:** It appears that both researchers and participants were aware of treatment allocation. Randomisation method not reported. Not clear whether groups were similar at baseline. Not all subjects analysed in the groups they were randomly allocated to. The turnover rates data came from the nursing care coordinators at each participating facility and was expressed as the number of people who had left the facility within the last fiscal year. Stress was measured with the Care Provider Questionnaire (Mahairas et al., 1990); self-esteem was measured with the Global Self-Esteem Scale (Rosenberg, 1965); coping style was measured with the Multidimensionality of Coping Scale (Endler & Parker, 1990). Authors suggest that the significant difference in coping style scores for between CNAs and LPNs by participation, indicated that empowering workers would have more of an effect for the workers with the lowest status. | | | | |
| A randomised controlled trial with data collected at baseline and 8 weeks later (immediately post-intervention). | The program proposed including CNAs and Licensed Practical Nurses (LPN) in interdisciplinary team meetings.  At the beginning of the intervention, all CNAs and LPNs were shown a short video describing the team approach in health care and team building.  The participants had to attend and contribute to interdisciplinary team meetings for 15-minutes each week for eight weeks. | n=64 nursing staff from n=10 long term care facilities in the Northeast, US; n=23 in the experimental group and n=23 in the control group; the sample included CNAs and LPNs (exact numbers not reported). | **Self-esteem**  No significant differences were found.  **Stress**  No significant differences were found.  **Coping style**  Subgroup analysis: A significant difference between CNAs who participated in the intervention and those who did not, when compared to LPNs who participated in the training and those who did not.  No other significant differences were found. | **Turnover rates**  No significant differences were found. |
| **Webb, H. (2003); Risk of bias: High;**  **Comment:** Low attrition, especially in the experimental arm (only 36 out of 59 completed the post-test). No randomisation. There were differences between groups at baseline. The comparison nursing home stopped their staff training six months prior to the study commencement. Turnover data was provided by the participating facilities. Turnover rate was calculated as the total number of termination in the preceding six months divided by the total number of CAN positions available; revolving door turnover rate was calculated as the total number of CAN terminations within six months of hire date within six-month period divided by the total number of CAN positions available; retention rate was calculated as the total number of CNAs with one year or more of service at the assessment time divided by the total number of CNA positions available; job satisfaction was measured with a modified version of the Nurse Assistant Assessment Survey (Bell, 1998); morale was measured with the Staff Morale Survey (Educatorsnet, 2001); empowerment was measured with the Employee Empowerment Questionnaire (Hayes, 2001). This study reports very good improvements in staff turnover over time and compared to the control group. However, the retention rate they reported for control group (107%) is not possible and likely an error. | | | | |
| A quasi-experimental non-equivalent control (comparison) group study. Data was collected at baseline, and six months later (immediately post-intervention). | This study combined two staff empowerment approaches, Character First and a Reward Program, to decrease CNA turnover. The study used Zimmerman’s (2000) empowerment theory as the framework.  The intervention was run over a period of six months. It focused on providing recognition of good character of CNAs and rewarding them for their continued service.  The programs were facilitated by the supervisors who attended one-day training seminar.  Formal recognition took place during monthly staff meetings. Three to five qualities (from a list of 49 character qualities) were given to each CNA, in order to formally recognise their good character. Also, during the monthly meetings, rewards were given to staff who were celebrating their service anniversary. The rewards were related to the employee’s career goal/dream. This was something that the employee always wanted to do in their career. The goal could be related to the employees’ will to, for example, pursue an engineer career. The goal didn’t have to be relevant to the nursing home. The aim of this approach was to show an interest in the employee and treating them as an asset, and not as an object of productivity. | n=98 CNAs from n=2 nursing homes in Rhode Island, US; n=47 CNAs in the experimental group and n=51 in the control group. | **Job satisfaction**  No significant changes were found.  **Staff morale**  No significant changes were found.  **Empowerment**  No significant changes were found. | **Turnover rate**  A significant decline in the experimental nursing home was reported.  **Revolving door turnover rate**  An improvement is reported, but no information provided regarding its statistical significance.  **Retention rate**  An improvement is reported, but no information provided regarding its statistical significance. |
| **Yeatts, D. et al.** **(2007); Risk of bias: High;**  **Comment:** No randomisation. Groups were not similar. The post-test period is reported as an average of 16 months for the intervention, and 17 months for the control group. The intervention was still running during post-test. The outcome measures were not previously validated. | | | | |
| A before and after study with a control group. Measurements were taken at baseline, and 16-17 months later (intervention still active). | The intervention focused on establishing empowered work teams of certified nursing assistants (CAN). The teams were organised by shift and service area (e.g. a wings of a nursing home). Establishing an empowered team involved training and orienting the CNAs, nurses and management of the facility.  The aim of the intervention was to empower the CNAs through involving them in management decisions related to CNAs work, reviewing residents’ health condition, reviewing new residents and their needs, and any other issues of concern to CNAs.  The team provided weekly written summaries of each team meeting to nurse management. The management reviewed the summaries and provided feedback to the teams.  The intervention involved also short stand-up meetings. These meetings were organised to address immediate concerns. For example, determining the best way to get all the residents to a planned activity, given a staff’s absence. | Certified nursing assistants in nursing homes in Texas; n= 314 to 353 CNAs in total; the exact number of participants in each group was not reported. | **Self-esteem**  No significant changes.  **Burnout**  No significant changes.  **General job satisfaction**  No significant changes.  **Empowerment**  The experimental group improved significantly over time in global empowerment, autonomy, meaningfulness, and competence. The difference over time was statistically significant between the groups for all but the competence subscale. | **Intention to quit**  No significant changes.  **Self-reported absenteeism**  No significant changes in the intervention group. Statistically significant increase in the control group.  **Turnover**  Significantly higher turnover in the control group post-intervention – however, baseline turnover unknown.  **Commitment**  No significant changes in the intervention group. Statistically significant decrease in the control group. |

## Table 4. Exercise and fitness interventions summary table.

| **Design** | **Intervention** | **Population** | **Psychosocial outcomes** | **Turnover-related outcomes** |
| --- | --- | --- | --- | --- |
| **Brox, J. et al. (2005); Risk of bias: Moderate;**  **Comment:** Randomisation, concealment and blinding methods not reported. Unclear how job satisfaction was measured. Sick leave utilisation was expressed as the total number of sick days in the preceding seven months. | | | | |
| A randomised controlled trial with data collected at baseline, and six months post implementation. | The study evaluated a fitness program intervention for nursing home employees. The program consisted of a weekly exercise session lasting for one hour.  The program was based on an aerobic fitness model and aimed to improve cardiovascular fitness, muscle strength and flexibility. It was delivered by experienced fitness instructors. Additionally, the participants were offered classes on physical exercise, nutrition and stress management. | n=129 nursing staff (nurses and NAs) from a nursing home in Norway; n=65 in the experimental group and n=64 in the control group. Exact number of NAs in the sample not reported. | **Job satisfaction**  No significant differences were found. | **Sick leave utilisation**  A significant increase in sick leave utilisation in the experimental group. |
| **Dreher, M., et al. (2019); Risk of bias: Very high;**  **Comments**: Pre-post design. No control group. Well reported. Used validated measures. Compassion satisfaction, burnout and posttraumatic stress were measured with the Professional Quality of Life Scale (Stamm, 2010). No definition of ‘retention’ was provided. Retention appeared to improve, but no statistical tests were performed. Retention rates from before the study period were used to compare them with the rates from after the intervention. Four months retention rates were provided for before and after. | | | | |
| A before and after study with no control group. Data was collected at baseline and one and three months later. | The study tested a 90-minute interactive educational presentation, which included 12 short YouTube video clips embedded in the presentation. The presentation addressed compassion fatigue awareness and  self-care skills. At the end of the presentation, participants were provided with a “Self-care Skills Toolbox”, which included a journal, pen, pedometer, stress ball, herbal tea and some reading materials. A healthy nutrition bag with healthy food items was also distributed to each participant. Take-away items served as a jump start for the participants to exercise self-care skills away from the workplace. | n=45 certified nursing assistants from a nursing home in Southeast, US. | **Compassion satisfaction**  No significant changes were found.  **Burnout**  Non-parametric statistically significant decrease over time. No other statistically significant changes reported.  **Stress**  Non-parametric statistically significant decrease over time. No other statistically significant changes reported. | **Retention**  No statistically significant changes reported. |
| **Flannery, K. et al. (2012); Risk of bias: High;**  **Comment:** Very small sample size. The groups were significantly different at baseline (work ability, effort, reward). No randomisation. Job satisfaction was measured with the Nursing Home Administrator Job Satisfaction Questionnaire (Castle, 2006); job stress was measured with the short version of the Effort, Reward and Imbalance Questionnaire (Leineweber et al., 2010); work ability and demands were measured with the Work Ability Index (Tuomi et al., 2003). | | | | |
| A before and after study design with a control group. Data was collected at baseline, and three and six months later (immediately post- and three months post-intervention). | This study evaluated a worksite health promotion program based on the theory of self-efficacy and the socioecological model. The aim of the intervention was to improve NAs job satisfaction, job stress, and work ability.  The program was delivered by a Master’s-prepared nurse, over a period of 3 months. It consisted of three main components: environmental assessment, initial education, and ongoing motivation.  Environmental assessment was a two-hour assessment of work factors affecting NAs health. Recommendations based on the assessment were made to the facility management.  Initial education was a 30-minute lecture on cardiovascular health, exercise and diet.  The third component, the ongoing motivation, was focused on motivating NAs to exercise, and reduce fat and salt intake. The program leader spent 40h/week focusing on this component in the first month, 16 h/week in the second and 8h/week in the third month of the project. Their role was to provide ongoing motivation and additional education to NAs. | n=39 NAs in n=2 long term care facilities in Maryland, US; n=24 NAs in the experimental group and n=15 in the control (education only) group. | **Job satisfaction**  No significant differences were found.  **Job stress**  No significant differences were found.  **Work ability and demands**  A significant treatment effect favouring the experimental group. | Not applicable. |
| **Young, C. et al. (2022); Risk of bias: Very high;**  **Comment:** No control group, before and after evaluation of an eight-week online intervention focusing on mindfulness practice. Stress was measured using the Depression Anxiety Stress Scale (Henry  & Crawford, 2005). Compassion satisfaction was measured using the Professional Quality of Life (Little &Rubin, 2002). | | | | |
| A before and after study with no control group. Measures were taken at baseline and immediately post completion of the intervention. | An 8-week online mindfulness-based intervention tailored for CNAs, modelled after the Mindfulness-Based Stress Reduction program by Kabat-Zinn. The educational content delivered each week was tailored to CNAs through examples relevant  to their working environment and tasks associated with their job. Each week, participants viewed a brief investigator-developed video that presented a concept for that week; formal and informal practice for the week was discussed and encouraged. It included meditation, mindful yoga, body scans, and practicing mindfulness when working with residents. The content was based on publicly available online resources. | N=19 certified nursing assistants in long-term care facilities in Texas, US | **Stress**  No statistically significant changes for the total score.  **Compassion satisfaction**  No statistically significant changes for the total score. | Not applicable |

# Appendix 5. Summary table of all included studies

## Table 5. Characteristics of the included studies.

| **Study** | **Intervention type** | **Design** | **Risk of bias** | **Setting and population**^1^ | **Intervention duration (D); Intervention focus (F); Core components (C)** | **Outcomes**^2^ | **Data availability** |
| --- | --- | --- | --- | --- | --- | --- | --- |
| Almquist 1981 | Knowledge-based | PrePost | Very high | USA  N=29, SW only  Nursing home | D: 6 weeks  F: general long-term care knowledge;  C: education sessions and interpersonal skills | Life satisfaction 0 | Y |
| Barbosa 2016 | Knowledge-based | RCT, placebo | Moderate | Portugal  N=53, SW only  Residential aged care facility | D: 2 months;  F: work-related stress and coping strategies;  C: education sessions, mentoring, relaxation and exercise | Perceived stress -  Burnout +  Job satisfaction 0 | Y |
| Beck 2013 | Knowledge-based | NRCT, non-placebo | High | Sweden  N=225, SW only  Residential aged care facility | D: a few weeks;  F: palliative care training;  C: education sessions | General job satisfaction -  Satisfaction with nursing care -/+  Strain -  Stress of conscience 0 | Y |
| Boersma 2017 | Interpersonal skills | NRCT, non-placebo | High | Netherlands  N=111, mixed sample  Nursing home | D: 9 months  F: communication skills with focus on dementia;  C: education sessions, interpersonal skills | Job satisfaction 0 | Y |
| Bright-Long 1990 | Knowledge-based | PrePost | Very high | USA  N=?, SW only  Nursing home | D: a few weeks;  F: understanding dementia;  C: education sessions | Burden 0 | N |
| Broughton 2011 | Interpersonal skills | NRCT, non-placebo | High | Australia,  N=52, mixed sample  Nursing home | D: 1 day;  F: communication skills with focus on dementia;  C: interpersonal skills; | Satisfaction 0 | Y |
| Brox 2005 | Self-care | RCT, non-placebo | Moderate | Norway  N=129, mixed sample  Nursing homes | D: 6 months;  F: Improve overall fitness;  C: relaxation and exercise | Job satisfaction 0  Sick leave utilisation - | Y |
| Catanzaro 1992 | Team-building | NRCT, non-placebo | High | USA  N=72, SW only  Nursing home | D: 2 months;  F: teamwork and problem solving;  C: team meetings | Turnover rates 0  Organisational commitment 0  Perceived influence 0  Satisfaction with service role 0  Satisfaction with influence 0  Satisfaction with higher order needs 0  Satisfaction with organisational policies 0 | Y |
| Cheng 2008 | Knowledge-based | PrePost | Very high | Taiwan  N=90, mixed sample  Nursing home | D: 3 weeks;  F: understanding dementia;  C: education sessions | Attitude +  Perceived self-efficacy +  Caregiving stress +  Job satisfaction 0  Health perception +  Quality of life 0 | N |
| Coogle 2006 | Knowledge-based | PrePost | Very high | USA  N=53, mixed sample  Long term care facility and in-home care | D: a few months;  F: understanding dementia;  C: education sessions, mentoring | Extrinsic job satisfaction +  Intrinsic job satisfaction 0  Career commitment - | Y |
| Covert 2007 | Interpersonal skills | PrePost | Very high | USA  N=97, SW only  Nursing home | D: 1 day;  F: communication skills with focus on dementia;  C: interpersonal skills | Turnover rates 0  Sick leave utilisation 0 | Y |
| Da Silva 2017 | Knowledge-based | PrePost | Very high | Brazil  N=25, mixed sample  Long term care facility | D: 6 weeks;  F: understanding dementia, work-related stress;  C: education sessions, communication skills | Burden 0  Depression 0  Anxiety 0  Quality of life 0 | Y |
| Dichter 2017 | Knowledge-based | NRCT, placebo | High | Germany  N=290, mixed sample  Nursing home | D: 18 months;  F: person-centred care, staff attitudes;  C: education sessions | Job satisfaction -  Burden -  Staff attitudes - | Y |
| Dreher 2019 | Self-care | PrePost | Very high | USA  N=45, SW only  Nursing home | D: 1 day;  F: compassion fatigue, coping strategies;  C: education sessions, self-care practice (relaxation, exercise, journaling) | Compassion satisfaction 0  Burnout +  Stress +  Retention rates 0 | Y |
| Finnema 2005 | Knowledge-based | RCT, placebo | Moderate | Netherlands  N=99, SW only  Nursing home | D: 7 months;  F: empathy;  C: education sessions, mentoring | Perceived work stress 0  Stress +  Absenteeism 0 | Y |
| Flannery 2012 | Self-care | NRCT, placebo | High | USA  N=39, SW only  Long term care facility | D: 3 months;  F: health promotion;  C: education sessions, exercise, mentoring, nutritional advice | Job satisfaction 0  Job stress 0  Work ability and demands + | Y |
| Fragala 2012 | Knowledge-based | PrePost | High | USA  N=24, mixed sample  Long term care facility | D: 3 months;  F: safe work environment;  C: education sessions, mentoring | Staff attitude + | N |
| Franzmann 2016 | Interpersonal skills | NRCT, placebo | High | Germany  N=116, mixed sample  Nursing home | D: a few months;  F: communication skills with focus on dementia;  C: mentoring, interpersonal skills | Mental stressors at work +  Occupational mental stress + | N |
| Fukuda 2018 | Knowledge-based | NRCT, non-placebo | Moderate | Japan  N=400, mixed sample  Long term care facility | D: 1 month;  F: understanding dementia;  C: education sessions | Burnout 0 | Y |
| Haberstroh 2011 | Interpersonal skills | NRCT, non-placebo | High | Germany  N=53, care worker group not specified  Nursing home | D: 7 weeks;  F: communication skills with focus on dementia;  C: interpersonal skills | Job stress + | N |
| Harman 1998 | Knowledge-based | NRCT, non-placebo | High | USA  N=11, SW only  Special dementia care unit | D: 2 weeks;  F: understanding dementia;  C: education sessions, mentoring, interpersonal skills | Turnover rates 0 | Y |
| Hegeman 2007A | Team-building | NRCT, non-placebo | High | USA  N=11, SW only  Nursing home | D: 3 months;  F: peer-mentoring to develop staff;  C: mentoring, education sessions, interpersonal skills | Retention rates + | Y |
| Hegeman 2007B | Team-building | PrePost | Very high | USA  N=?, SW only  Nursing home | D: 6 months;  F: peer-mentoring to develop staff;  C: mentoring, education sessions, interpersonal skills | Retention rates - | Y |
| Howe 2014 | Team-building | PrePost | Very high | USA  N=15, mixed sample  Long term care facilities | D: 1 month;  F: group debriefing;  C: team meetings | Quality of work life +  Intention to quit + | N |
| Hsieh 2009 | Knowledge-based | NRCT, non-placebo | High | Taiwan  N=112, mixed sample  Nursing home | D: 2 months;  F: understanding aging, work-related stress and coping strategies  C: education sessions | Caregiver stress 0 | Y |
| Inker 2021 | Knowledge-based | PrePost | Very high | USA  N=250, mixed sample  Long term care facilities | D: 52 weeks  F: provision of person-centred care  C: education sessions (online) | Job satisfaction +  Staff attitude + | Y |
| Isaia 2011 | Knowledge-based | PrePost | Very high | Italy  N=50, mixed sample  Nursing home | D: 8 months;  F: understanding aging, work-related stress and coping strategies  C: education sessions | Stress -/+ | N |
| Jung 2020 | Knowledge-based | NRCT, non-placebo | Very high | South Korea  N=50, SW only  Long term care facilities | D: 6 weeks  F: provision of function-focused care  C: education sessions | Job satisfaction 0  Self-esteem 0 | Y |
| Kuske 2009 | Knowledge-based | RCT, placebo and non-placebo | Low | Germany  N=96, mixed sample  Nursing home | D: 3 months;  F: understanding dementia;  C: education sessions, interpersonal skills | Burnout 0  Health complaints 0 | Y |
| Li-Yu 2005 | Knowledge-based | NRCT, non-placebo | High | Taiwan  N=35, SW only  Long term care facility | D: 3 months;  F: empowerment;  C: education sessions | Work stressors - | N |
| MacDonald 2007 | Knowledge-based | PrePost | Very high | USA  N=753, mixed sample  Long term care facility | D: a few months;  F: general long-term care knowledge;  C: education sessions | Turnover rates 0 | Y |
| Mackenzie 2003 | Knowledge-based | NRCT, non-placebo | High | Canada  N=41, mixed sample  Long term care facility | D: 1 month;  F: managing challenging work situations;  C: education sessions | Self-efficacy +  Burnout +  Satisfaction with teamwork and co-workers 0 | Y |
| McCallion 1999 | Interpersonal skills | RCT, non-placebo | Moderate | USA  N=88, SW only  Nursing home | D: a few months;  F: communication skills with focus on dementia;  C: interpersonal skills | Turnover rates + | Y |
| Noel 2000 | Knowledge-based | NRCT, non-placebo | High | USA  N=?, care worker group not specified  Long term care facility | D: 1 year;  F: general long-term care knowledge, rewards for good performance;  C: education sessions, mentoring, rewards | Turnover rates +  Absenteeism 0 | N |
| Passalacqua 2012 | Interpersonal skills | PrePost | Very high | USA  N=26, SW only  Long term care facility | D: 1 month;  F: communication skills with focus on person centredness;  C: interpersonal skills | Happiness 0  Burnout 0 | Y |
| Petterson 2006 | Team-building | PrePost | Very high | Sweden  N=200, mixed sample  Nursing home and in-home care | D: 18 months;  F: peer-mentoring to develop staff;  C: mentoring, education sessions | Workload 0  Staff resources -  Health and well-being -  Health resources 0 | N |
| Pillemer 2003 | Interpersonal skills | RCT, non-placebo | Moderate | USA  N=655, mixed sample  Nursing home | D: 1 day;  F: communication skills with resident's family;  C: interpersonal skills | Depressive symptomatology 0  Burnout 0  Intention to quit + | Y (some) |
| Pillemer 2008 | Team-building | RCT, non-placebo | Moderate | USA  N=762, mixed sample  Nursing home | D: 1 year;  F: peer-mentoring to improve retention;  C: mentoring, education sessions | Job satisfaction 0  Stress 0  Turnover rates +  Job commitment + | Y (some) |
| Resnick 2009 | Knowledge-based | RCT, placebo | Moderate | USA  N=556, SW only  Nursing home | D: 1 year;  F: restorative care approach;  C: education sessions, mentoring | Job satisfaction + | Y |
| Robison 2007 | Interpersonal skills | RCT, non-placebo | Moderate | USA  N=384, mixed sample  Nursing home | D: 1 day;  F: communication skills with focus on dementia;  C: education sessions, interpersonal skills | Depression 0  Burnout 0  Job satisfaction 0  Job stress 0  Intention to quit 0 | N |
| Schrijnemaekers 2003 | Interpersonal skills | RCT, non-placebo | High | Netherlands  N=300, care worker group not specified  Homes for elderly | D: 1 year;  F: communication skills with focus on dementia;  C: education sessions, mentoring, interpersonal skills | Job satisfaction +  Burnout +  Work situation 0  Sick leave utilisation 0 | N |
| Sprangers 2015 | Interpersonal skills | NRCT, non-placebo | High | Netherlands  N=24, SW only  Nursing home | D: a few weeks;  F: communication skills with focus on dementia;  C: interpersonal skills | Job satisfaction 0  Caregiver distress + | Y |
| Tannazzo 2008 | Knowledge-based | PrePost | Very high | USA  N=3-1, SW only  Nursing home | D: 1 month;  F: understanding dementia;  C: education sessions | Job satisfaction 0  Intrinsic job satisfaction + | Y |
| Teri 2005 | Interpersonal skills | RCT, non-placebo | High | USA  N=25, SW only  Assisted living facility | D: 2 months;  F: communication skills with focus on dementia;  C: interpersonal skills, education sessions | Job satisfaction 0 | N |
| Torsney 2000 | Team-building | RCT, non-placebo | High | USA  N=64, mixed sample  Long term care facility | D: 2 months;  F: including SWs in team meetings;  C: team meetings | Self-esteem 0  Stress 0  Coping style +  Turnover rates 0 | Y |
| Tynan 1984 | Knowledge-based | PrePost | Very high | USA  N=?, care worker group not specified  Long term care facility | D: a few weeks;  F: general long-term care knowledge;  C: education sessions, mentoring | Turnover rates 0 | N |
| Webb 2003 | Team-building | NRCT, non-placebo | High | USA  N=98, SW only  Nursing home | D: 6 months;  F: rewards for good service;  C: rewards | Job satisfaction 0  Staff morale 0  Empowerment 0  Turnover rates +  Retention rates 0 | Y |
| Wells 2000 | Knowledge-based | RCT, non-placebo | High | Canada  N=?, care worker group not specified  Nursing home | D: a few months;  F: abilities-focused care approach;  C: education sessions | Caregiver stress 0 | Y |
| Yeatts 2007 | Team-building | NRCT, non-placebo | High | USA  N=353, SW only  Nursing home | D: 17 months;  F: involving SWs in making decisions;  C: team meetings | Self-esteem 0  Burnout 0  Job satisfaction 0  Empowerment +  Intention to quit 0  Absenteeism 0  Turnover rates 0  Job commitment 0 | Y |
| Young 2022 | Self-care | PrePost | Very high | USA  N=19, SW only  Long term care facility | D: 8 weeks  F: mindfulness in caregiving  C: education sessions, relaxation practice | Stress 0  Compassion satisfaction 0 | Y |
| Zimmerman 2010 | Knowledge-based | RCT, non-placebo | Moderate | USA  N=662, mixed sample  Long term care facility | D: 6 weeks;  F: understanding dementia;  C: education sessions, interpersonal skills | Work stress -  Job satisfaction +  Role recognition 0 | Y |
| ^1^ ‘?’ – not reported.  2 ‘+’ – some statistically significant positive changes reported; ‘-‘ some statistically significant negative changes reported; ‘0’ – no statistically significant changes reported. | | | | | | | |
|  | | | | | | | |

**References**

1. Almquist E, Stein S, Weiner A, Linn MW. Evaluation of Continuing Education for Long‐Term Care Personnel: Impact upon Attitudes and Knowledge. Journal of the American Geriatrics Society. 1981;29(3):117-22.

2. Barbosa A, Nolan M, Sousa L, Marques A, Figueiredo D. Effects of a Psychoeducational Intervention for Direct Care Workers Caring for People with Dementia. American Journal of Alzheimer's Disease and other Dementias. 2016;31(2):144-55.

3. Beck I, Jakobsson U, Edberg AK. Applying a palliative care approach in residential care: Effects on nurse assistants' work situation. Palliative and Supportive Care. 2013;13(3):543-53.

4. Boersma P, Dröes RM, Lissenberg-Witte BI, van Meijel B, van Weert JCM. Does working with the Veder Contact Method influence the job satisfaction of caregivers? A non-randomized controlled trial in nursing homes for people with dementia. International Psychogeriatrics. 2017:1-16.

5. Bright-Long LE. Easing the burden of caregiving for the paraprofessionals. Advances in Experimental Medicine and Biology. 1990;282:121-6.

6. Broughton M, Smith ER, Baker R, Angwin AJ, Pachana NA, Copland DA, et al. Evaluation of a caregiver education program to support memory and communication in dementia: A controlled pretest–posttest study with nursing home staff. International Journal of Nursing Studies. 2011;48(11):1436-44.

7. Brox JI, Froøystein O. Health-related quality of life and sickness absence in community nursing home employees: Randomized controlled trial of physical exercise. Occupational Medicine. 2005;55(7):558-63.

8. Catanzaro D. The impact of an employee involvement program on service quality in a nursing home organization: OLD DOMINION UNIVERSITY; 1992.

9. Cheng W. Adapt and evaluate an education program on behavioral and psychological symptoms of dementia for nursing caregivers in Taiwan long-term care facilities: University of Minnesota; 2008.

10. Coogle CL, Head CA, Parham IA. The long-term care workforce crisis: Dementia-care training influences on job satisfaction and career commitment. Educational Gerontology. 2006;32(8):611-31.

11. Covert BK. The effects of communication and interpersonal skills training on the job satisfaction of certified nursing assistants in nursing home facilities: Capella University; 2007.

12. da Silva Serelli L, Reis RC, Laks J, de Padua AC, Bottino CM, Caramelli P. Effects of the Staff Training for Assisted Living Residences protocol for caregivers of older adults with dementia: A pilot study in the Brazilian population. Geriatrics and Gerontology International. 2017;17(3):449-55.

13. Dichter MN, Trutschel D, Schwab CGG, Haastert B, Quasdorf T, Halek M. Dementia care mapping in nursing homes: effects on caregiver attitudes, job satisfaction, and burnout. A quasi-experimental trial. International Psychogeriatrics. 2017:1-14.

14. Dreher MM, Hughes RG, Handley PA, Tavakoli AS. Improving Retention Among Certified Nursing Assistants Through Compassion Fatigue Awareness and Self-Care Skills Education. Journal of Holistic Nursing. 2019;37(3):296-308.

15. Finnema E, Dröes R, Ettema T, Ooms M, Adèr H, Ribbe M, et al. The effect of integrated emotion-oriented care versus usual care on elderly persons with dementia in the nursing home and on nursing assistants: a randomized clinical trial. International Journal of Geriatric Psychiatry. 2005;20(4):330-43.

16. Flannery K, Resnick B, McMullen TL. The impact of the worksite heart health improvement project on work ability: A pilot study. Journal of Occupational and Environmental Medicine. 2012;54(11):1406-12.

17. Fragala G. Creating safer environments for long-term care staff and residents. Annals of Long Term Care. 2012;20(2):8p-p.

18. Franzmann J, Haberstroh J, Pantel J. Train the trainer in dementia care: A program to foster communication skills in nursing home staff caring for dementia patients. Zeitschrift fur Gerontologie und Geriatrie. 2016;49(3):209-15.

19. Fukuda K, Terada S, Hashimoto M, Ukai K, Kumagai R, Suzuki M, et al. Effectiveness of educational program using printed educational material on care burden distress among staff of residential aged care facilities without medical specialists and/or registered nurses: Cluster quasi-randomization study. Geriatrics and Gerontology International. 2018;18(3):487-94.

20. Haberstroh J, Franzmann J, Krause K, Pantel J. The influence of social competence on occupational stress of nursing home staff caring for dementia patients. Psychology of Burnout: Predictors and Coping Mechanisms2011. p. 199-214.

21. Harman BJ. The effects of a paraprofessional preceptor program for certified nursing assistants in dementia special care units: Saint Louis University; 1998.

22. Hegeman C, Hoskinson D, Munro H, Maiden P, Pillemer K. Peer mentoring in long-term care: rationale, design, and retention. Gerontology and Geriatrics Education. 2007;28(2):77-90.

23. Howe EE. Empowering certified nurse's aides to improve quality of work life through a team communication program. Geriatric Nursing (New York, NY). 2014;35(2):132-6.

24. Hsieh HF, Wang JJ, Yen M, Liu TT. Educational support group in changing caregivers' psychological elder abuse behavior toward caring for institutionalized elders. Advances in Health Sciences Education. 2009;14(3):377-86.

25. Inker J, Jensen C, Barsness S, Stewart MM. Implementing Microlearning in Nursing Homes: Implications for Policy and Practice in Person-Centered Dementia Care. Journal of Applied Gerontology. 2021;40(9):1062-70.

26. Isaia G, Astengo M, Isaia GC, Bo M, Cappa G, Mondino S, et al. Stress in professional care-givers working with patients with dementia: A hypothesis-generating study. Aging Clinical and Experimental Research. 2011;23(5-6):463-9.

27. Jung D, De Gagne JC, Lee M, Lee H. The effect of function-focused care on long-term care workers in South Korea. Geriatric Nursing. 2020;41(5):629-34.

28. Kuske B, Luck T, Hanns S, Matschinger H, Angermeyer MC, Behrens J, et al. Training in dementia care: A cluster-randomized controlled trial of a training program for nursing home staff in Germany. International Psychogeriatrics. 2009;21(2):295-308.

29. Li-yu W, Yin TJC, I-chuan L. The effectiveness of empowering in-service training programs for foreign nurse aides in community-based long-term care facilities. Public Health Nursing. 2005;22(2):147-55.

30. MacDonald CJ, Walton R. E-learning education solutions for caregivers in long-term care (LTC) facilities: new possibilities. Education for Health. 2007;20(3):85.

31. Mackenzie CS, Peragine G. Measuring and enhancing self-efficacy among professional caregivers of individuals with dementia. American Journal of Alzheimer's Disease and other Dementias. 2003;18(5):291-9.

32. McCallion P, Toseland RW, Lacey D, Banks S. Educating nursing assistants to communicate more effectively with nursing home residents with dementia. Gerontologist. 1999;39(5):546-58.

33. Noel MA, Pearce GL, Metcalf R. Front line workers in long-term care: the effect of educational interventions and stabilization of staffing ratios on turnover and absenteeism. Journal of the American Medical Directors Association. 2000;1(6):241-7.

34. Passalacqua SA, Harwood J. VIPS Communication Skills Training for Paraprofessional Dementia Caregivers: An Intervention to Increase Person-Centered Dementia Care. Clinical Gerontologist. 2012;35(5):425-45.

35. Petterson IL, Donnersvärd HÅ, Lagerström M, Toomingas A. Evaluation of an intervention programme based on empowerment for eldercare nursing staff. Work and Stress. 2006;20(4):353-69.

36. Pillemer K, Suitor JJ, Henderson Jr CR, Meador R, Schultz L, Robison J, et al. A cooperative communication intervention for nursing home staff and family members of residents. Gerontologist. 2003;43(SPEC. ISS. 2):96-106.

37. Pillemer K, Meador R, Henderson Jr C, Robison J, Hegeman C, Graham E, et al. A facility specialist model for improving retention of nursing home staff: Results from a randomized, controlled study. Gerontologist. 2008;48(SPEC. ISS. 1):80-9.

38. Resnick B, Gruber-Baldini AL, Galik E, Pretzer-Aboff I, Russ K, Hebel JR, et al. Changing the philosophy of care in long-term care: Testing of the restorative care intervention. Gerontologist. 2009;49(2):175-84.

39. Robison J, Curry L, Gruman C, Porter M, Henderson Jr CR, Pillemer K. Partners in caregiving in a special care environment: Cooperative communication between staff and families on dementia units. Gerontologist. 2007;47(4):504-15.

40. Schrijnemaekers VJJ, Van Rossum E, Candel MJJM, Frederiks CMA, Derix MMA, Sielhorst H, et al. Effects of emotion-oriented care on work-related outcomes of professional caregivers in homes for elderly persons. Journals of Gerontology - Series B Psychological Sciences and Social Sciences. 2003;58(1):S50-S7.

41. Sprangers S, Dijkstra K, Romijn-Luijten A. Communication skills training in a nursing home: Effects of a brief intervention on residents and nursing aides. Clinical Interventions in Aging. 2015;10:311-9.

42. Tannazzo T, Breuer L, Williams S, Andreoli NA. A dementia training program to benefit certified nurse assistant satisfaction and nursing home resident outcomes. Alzheimer's Care Today. 2008;9(4):221-9.

43. Teri L, Huda P, Gibbons L, Young H, Van Leynseele J. STAR: A dementia-specific training program for staff in assisted living residences. Gerontologist. 2005;45(5):686-93.

44. Torsney KM. Empowering lower status workers in long-term care: The impact of including CNAs and LPNs in interdisciplinary team meetings on their levels of turnover, stress, coping, and self-esteem: Columbia University; 2000.

45. Tynan C, Witherell J. Good orientation cuts turnover. Geriatric Nursing. 1984;5(3):173-5.

46. Webb HS. Testing of an intervention to decrease certified nursing assistant (CNA) turnover in a nursing home: University of Rhode Island; 2003.

47. Wells DL, Dawson P, Sidani S, Craig D, Pringle D. Effects of an abilities-focused program of morning care on residents who have dementia and on caregivers. Journal of the American Geriatrics Society. 2000;48(4):442-9.

48. Yeatts DE, Cready CM. Consequences of empowered CNA teams in nursing home settings: A longitudinal assessment. Gerontologist. 2007;47(3):323-39.

49. Young CC, Kesler S, Walker VG, Johnson A, Harrison TC. An Online Mindfulness-based Intervention for Certified Nursing Assistants in Long-term Care. Journal of holistic nursing : official journal of the American Holistic Nurses' Association. 2022:8980101221105709.

50. Zimmerman S, Mitchell CM, Reed D, Preisser JS, Fletcher S, Beeber AS, et al. Outcomes of a Dementia Care training program for staff in nursing homes and residential care/assisted living settings. Alzheimer's Care Today. 2010;11(2):83-99.

1. The MBI measure for burnout provides 269 ratings in three domains (emotional exhaustion, depersonalization and personal accomplishment). It is not recommended they are combined.( Maslach C, Jackson S, Leiter M. Maslach Burnout Inventory Manual. Palo Alto, CA: Consulting 523 Psychologists Press;; 1996.) [↑](#footnote-ref-1)
